# Tuberculosis Drug Discovery: A Decade of Hit Assessment for Defined Targets

**DOI:** 10.3389/fcimb.2021.611304

**Published:** 2021-03-15

**Authors:** Sangmi Oh, Lena Trifonov, Veena D. Yadav, Clifton E. Barry, Helena I. Boshoff

**Affiliations:** Tuberculosis Research Section, Laboratory of Clinical Immunology and Microbiology, National Institute of Allergy and Infectious Diseases (NIAID), National Institutes of Health (NIH), Bethesda, MD, United States

**Keywords:** tuberculosis, antitubercular agent, structure-activity relationship, drug target, cell wall, respiration

## Abstract

More than two decades have elapsed since the publication of the first genome sequence of *Mycobacterium tuberculosis* (*Mtb*) which, shortly thereafter, enabled methods to determine gene essentiality in the pathogen. Despite this, target-based approaches have not yielded drugs that have progressed to clinical testing. Whole-cell screening followed by elucidation of mechanism of action has to date been the most fruitful approach to progressing inhibitors into the tuberculosis drug discovery pipeline although target-based approaches are gaining momentum. This review discusses scaffolds that have been identified over the last decade from screens of small molecule libraries against *Mtb* or defined targets where mechanism of action investigation has defined target-hit couples and structure-activity relationship studies have described the pharmacophore.

## Introduction

The diversity of candidates in the tuberculosis (TB) drug discovery pipeline has grown over the last decade (https://www.newtbdrugs.org). However, the attrition rate for drug discovery is very high and continued efforts are needed. Phenotypic whole-cell screens have historically provided the best successes from hit to pre-clinical or clinical candidate. Recently, however, a better understanding of target vulnerability in the context of human pathogenesis has paved the way for target-based screens where hits that engage novel proteins may provide a future generation of scaffolds with promise (Kana et al., 2014). In this review, we have focused on scaffolds that have been identified from screens of small molecule libraries against whole-cells of *Mtb* or defined protein targets where experimental evidence identified or supported a specific target and the structure-activity relationships (SAR) for these series. Several of the series in this review inhibit processes that have already been validated either by established clinical efficacy of existing antitubercular drugs that target these pathways or in animal models to the extent that inhibitors for enzymes in the pathway have progressed to clinical evaluation (https://www.newtbdrugs.org/pipeline/clinical). Other series engage targets with more limited evidence of target vulnerability in the context of human disease. Nevertheless, despite genetic or even chemical proof of target vulnerability, the mechanism of protein inhibition may ultimately be responsible for the observed phenotype. Thus, genetic depletion informs us of the consequences of target depletion while chemical inhibition may result in metabolomic alternations that are not always predictable based on function of the enzyme in normal metabolism. In addition, the availability of metabolites with the potential to rescue cells from target inhibition is a further confounder to predicting target vulnerability (Evans et al., 2016; Park et al., 2017; Singh et al., 2017; Beites et al., 2019; Wang et al., 2019).

This review will not discuss series based on known scaffolds (prior to 2010) against *Mtb* targets, series identified by fragment-based drug discovery or hits identified by virtual screening, scaffold morphing, hopping or hybridization. We will highlight the important aspects of the SAR, target validation as well as efficacy studies while acknowledging obvious liabilities of certain series.

## Antitubercular Agents Regulating Mycolylarabinogalactan Peptidoglycan (mAGP) Assembly

### DprE1

DprE1 (decaprenylphosphoryl-β-D-ribofuranose 2’-oxidase) is the FAD-dependent epimerase that converts decaprenyl-phospho-ribose to the decaprenyl-phospho-2’-keto-D-arabinose intermediate which is channeled to DprE2 for reduction to decaprenyl-phospho-arabinose. The decaprenyl-phospho-arabinose is the building block of the arabinan component of the mycolyl-arabinogalactan component of the mycobacterial cell wall. DprE1 catalytic activity is essential for mycobacterial growth (Sassetti and Rubin, 2003; Riccardi et al., 2013).

Makarov and co-workers discovered the benzothiazinones as highly potent inhibitors of *Mtb* growth based on covalent inactivation of DprE1 (Makarov et al., 2009). A safer and more effective analog of this benzothiazinone, PBTZ169 (Macozinone), recently completed phase 1 clinical trials (ClinicalTrials.gov: NCT04150224) (Makarov et al., 2009; Makarov et al., 2014), showing only mild side-effects in both single and multi-dose safety assessments. A concern with the PBTZ169 is that the reactive head-group may covalently modify human targets although no evidence for this has been found to date. A variety of metabolites have been identified in the first in human studies (Spaggiari et al., 2019). A non-covalent azaindole-based DprE1 inhibitor finished phase I clinical trials in 2018 but has not yet reported results (ClinicalTrials.gov: NCT03199339).

#### 4-Aminoquinolone Piperidine Amides

The aminoquinolone scaffold was identified by whole-cell screening of a library consisting of 320K compounds from AstraZeneca with the initial hit, compound **1** (**Table 1**), showing excellent lead-like properties including good solubility, activity against replicating *Mtb* (MIC_80_ = 6.3 µM), and a good secondary pharmacological profile (Naik et al., 2014). Analysis of mutants and enzyme assays confirmed DprE1 as the target. Despite the potent *in vitro* cidality of the compounds, the intracellular activity against *Mtb* growing in macrophages was modest and not dose-dependent.

**Table 1:** Antitubercular agents inhibiting mycolylarabinogalactan peptidoglycan (mAGP) assembly.

No.	Target	Scaffold	SAR plan from hit	Most advanced analogue	*In vivo* efficacy	Ref.
1.1	DprE1	4−Aminoquinolone piperidine amides	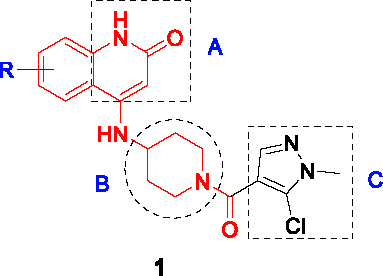	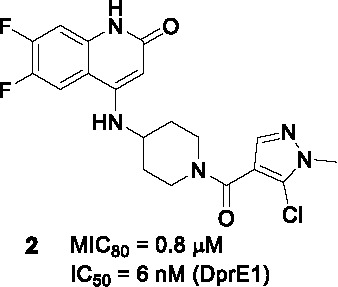	N/D*^a^*	(Naik et al., 2014)
1.2		Pyrazolopyridones	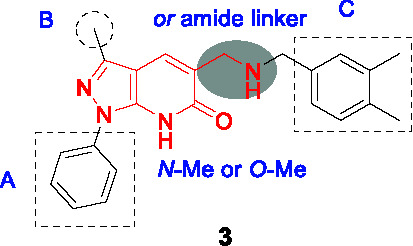	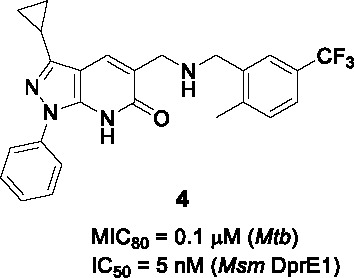	N/D	(Panda et al., 2014)
1.3		2-Carboxy quinoxalines	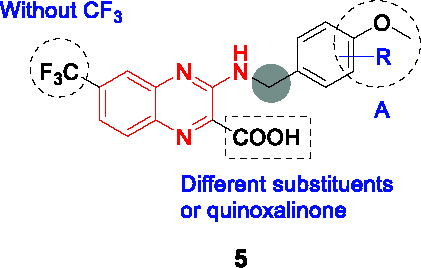	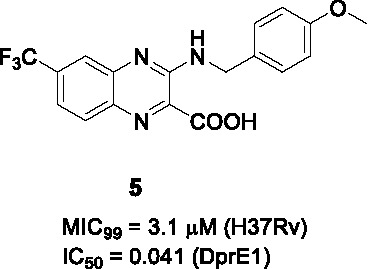	N/D	(Neres et al., 2015)
1.4		5-Hydroxy pyrimidinediones	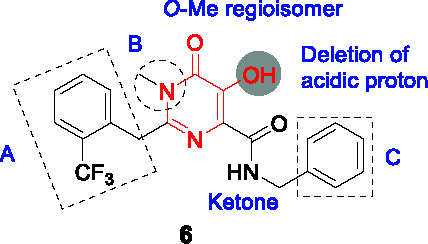	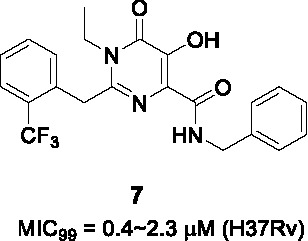	N/D	(Oh et al., 2018)
2.1	MmpL3	Adamantyl ureas	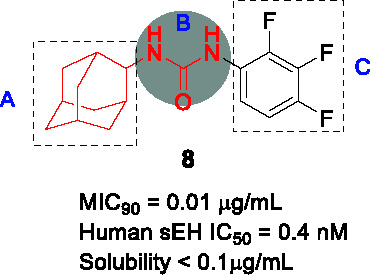	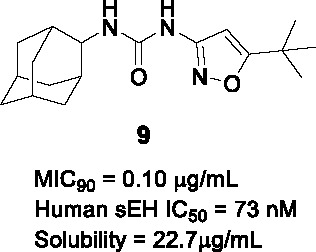	N/D	(Brown et al., 2011; North et al., 2013)
2.2		Indolecarboxamides	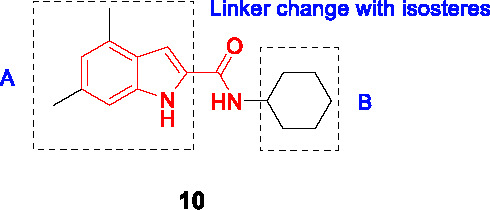	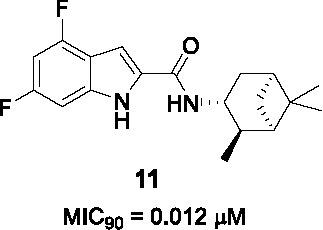	Yes*^b^*	(Onajole et al., 2013; Stec et al., 2016)
2.3			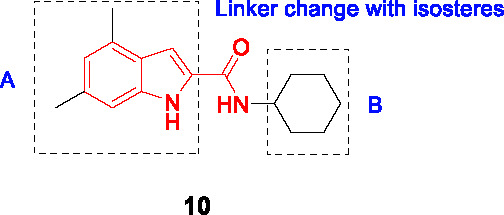	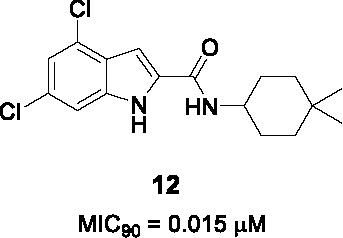	Yes*^b^*	(Kondreddi et al., 2013)
2.4		Tetrahydropyrazolo pyrimidine carboxamide	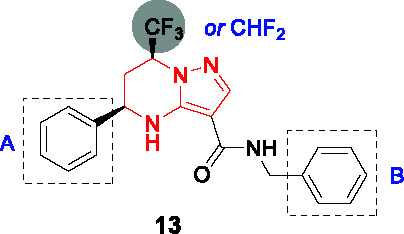	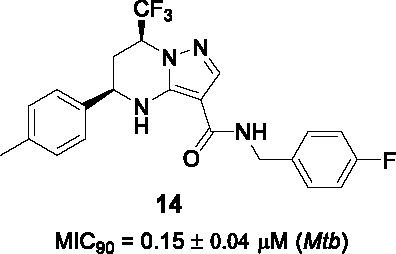	Yes*^b^*	(Remuinan et al., 2013; Yokokawa et al., 2013)
2.5		Spirocycles	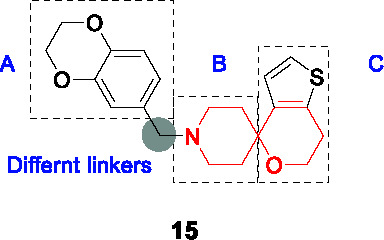	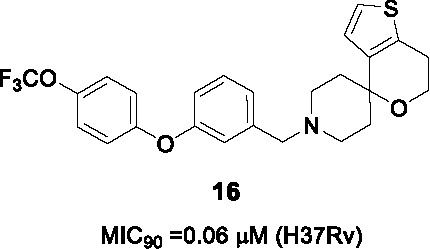	Yes*^b^*	(Remuinan et al., 2013; Guardia et al., 2018)
3.1	MurI	Benzoxazoles	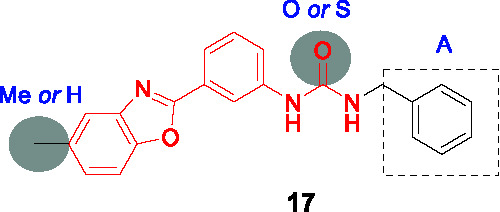	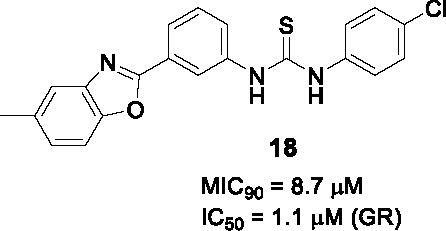	Yes*^c^*	(Malapati et al., 2018)

^a^N/D, not determined; ^b^Yes, active in an acute TB infection mouse model; ^c^ Yes, active in Mycobacterium marinum infected adult zebrafish model.

The SAR was initially guided by *in silico* docking studies based on the presumed binding mode of the aminoquinolone scaffold to DprE1. However, the computational predictions to increase van der Waals interactions in the quinolone (A) moiety only yielded 6- and 6,7- fluorinated analogs with increased potency leading to the most advanced analog (**2**, MIC_80_ = 0.8 µM). Modifications on the piperidine (B) resulted in the stereoselective azabicyclo[3.2.1]octanyl ring with 10-fold improvement in antitubercular activity observed for this linker. While the stereospecific piperidine or octanyl derivatives showed improved potency, cross resistance with the DprE1 C387S *Mtb* mutant as well as DprE1-overexpressing strains was lost despite excellent enzyme inhibition data, suggesting potential off-target effects. Assessment of the pyrazole ring in C showed that the chlorine substituent was important whereas replacing the methyl with a bulky isopropyl resulted in loss of activity. Replacement of the pyrazole with an isoxazole was tolerated. The *in vitro* DMPK and safety profiles of some 4-aminoquinolone piperidine amide analogs including **2** were promising but showed poor oral exposure in mice preventing further evaluation of chemotherapeutic efficacy in the animal model.

Activity-guided SAR revealed some analogs modified in the piperidine part with stereoselective bulky heterocycles that displayed excellent enzymatic and whole-cell activity but different cross-resistance against *Mtb* mutants compared to **1**. Thus, despite improvement of potency against both the enzyme and whole cells, the off-target effects of some analogs need to be better understood.

#### Pyrazolopyridones

The pyrazolopyridone scaffold was identified from a whole-cell screening of an AstraZeneca library against *Mtb* followed by assessment of cidality and cytotoxicity (Panda et al., 2014). Although the initial hit (**3**, **Table 1**) had weak whole-cell activity (MIC_80_ = 25 µM), SAR exploration led to a 250-fold improvement in potency. Like the aminoquinolones (Naik et al., 2014), the pyrazolopyridone series were found to be cidal against replicating *Mtb*, had no activity against non-replicating cells, modest activity against *Mtb* growing in macrophages with over-expression of DprE1 conferring resistance. The same mutations that conferred aminoquinolone resistance conferred resistance to this series with DprE1 inhibition further confirmed by enzymology.

The SAR exploration with 22 synthetic analogs was driven by whole-cell activity. The modification of the pyrazolopyridone core with *O*-Me or *N*-Me resulted in complete loss of activity, suggesting important hydrogen bonding of the core in the protein binding site. Although methylation of secondary amine linker improved antitubercular activity, this was not applied to optimize this series due to lipophilicity concerns. Limited modifications in A confirmed phenyl as preferred at this position, and changing the methyl group in B to cyclopropyl improved activity. Importantly, an 8-fold enhancement in activity was obtained by replacing a *m*-Me with a trifluoromethyl group in C, suggesting that the *m*-CF_3_ was making important contacts to DprE1 active site residues. Replacement of the phenyl with a pyridine moiety in C in order to decrease lipophilicity led to a moderate decrease in potency. MIC and lipophilicity were tightly correlated in this series. Testing against the enzyme showed a good correlation between whole cell activity and enzyme inhibition. Compound **4** stood out as a representative analog with highly improved antitubercular activity (MIC_80_ = 0.1 µM, MBC < 0.5 µM), but suffered from poor solubility, high metabolic turnover in rat hepatocytes, and high plasma protein binding. Thus, further optimization of this series will be required in order to perform *in vivo* validation of their chemotherapeutic efficacy.

#### 2-Carboxyquinoxalines

Compound **5** (**Table 1**), a 2-carboxyquinoxaline scaffold, was identified in a phenotypic screen of a 266-member quinoxaline library against *Mtb* (Neres et al., 2015). The best analogs including **5** were selected based on low micromolar MIC values and activity against *Mtb* growing in macrophages (MIC_99_ = 3.1 µM, IC_50_ = 2.5 µM, respectively) and modest selectivity index against HepG2 cells.

The compounds were associated with a high frequency of resistance mapping to inactivating mutations in a transcriptional repressor encoded by *Rv3405c* which controls transcription of *Rv3406* encoding an iron- and α-ketoglutarate-dependent sulfate ester dioxygenase. Inactivation of the Rv3405c transcriptional repressor resulted in upregulation of expression of the dioxygenase which decarboxylates the 2-carboxyquinoxalines to an inactive keto derivative. In the absence of the dioxygenase, resistance-conferring mutations mapped to *dprE1* where mutation mapping defined an overlapping binding site to the benzothiazinones which was validated by co-crystal structure. The prediction of DprE1 as the target was further confirmed by DprE1 over-expression (hereby conferring increased resistance) as well enzyme kinetics showing noncompetitive inhibition of DprE1 by 2-carboxyquinoxalines.

A SAR study of **5** was performed with a limited focused library, especially to improve whole-cell activity and DprE1 inhibition, while avoiding metabolic inactivation by Rv3406 (Sogi et al., 2013). Based on the assumption that quinoxaline core was essential, the focus was on fine-tuning of the phenyl ring (A) and limited exploration of other substituents. The carboxylate group was critical for dioxygenase activity, but it was also critical for antitubercular activity because the replacement with methyl or carboxamide resulted in loss of whole-cell activity and negligible inhibition of DprE1. Although esters at this position had been found to be active in whole-cell assays, these did not inhibit DprE1 suggesting that the esters are hydrolyzed by esterases or spontaneously in the growth medium. The removal of trifluoromethyl group from the core was not tolerated. Interestingly, most analogs modified in the A ring with different substituents retained enzymatic activity against DprE1 but only some of them, especially *p*-OMe or *p*-Cl benzyl compounds improved whole-cell activity. This result reiterated the importance of correlating target inhibition with whole-cell activity since the latter reflects the additional factors effected by compound uptake, efflux and modification. Overall, the SAR study showed limited capacity for improvement with steep SAR based on the limited number of compounds reported. None of the analogs was superior to hit **5** based on MIC as well as DprE1 inhibition (IC_50_ = 0.041 µM). The availability of X-ray crystallography data of DprE1 co-crystalized with this 2-carboxyquinoxaline series, allows for the possibility of further structure-based analogue design.

#### 5-Hydroxy pyrimidinediones

The pyrimidinedione scaffold was identified by HTS of a library from St. Jude Children’s Research Hospital comprised of 6,207 molecules containing the pyrimidinedione substructure. The rationale for screening against pyrimidinediones was based on the favorable druglike properties of this scaffold which is found in a variety of FDA approved drugs including Raltegravir, many of which have been found to inhibit Mg^2+^-dependent enzymes based on their Mg^2+^ chelation within the active site of their targets (Oh et al., 2018). The *N*-alkyl-5-hydroxypyrimidinone carboxamide hit **6** (**Table 1**) was selected based on its more favorable physicochemical properties but no evidence of Mg^2+^-dependence was found with the resistance conferring mutations mapping to *dprE1* which was further confirmed by DprE1 under-expression studies.

To explore the SAR, a library of 17 novel compounds was synthesized and evaluated for whole-cell activity to understand the functional contributions of each moiety in the entire structure. The phenyl ring (A) with the *o*-CF_3_ substitution was found to be most favorable for activity. A methyl substitution in the benzylic position was detrimental for activity. Replacement of methyl with ethyl in B improved activity, while its deletion was not tolerated. Deletion of the acidic proton on the 5-hydroxy group or methyl protection led to complete loss of activity. Replacement of the benzyl in C with phenyl reduced potency although 2-picolyl was tolerated. Fluorine substitution at the *ortho* position in C was favored above that in the *para* position. Methylation at the amide or benzylic position led to loss of activity. The best compound from this SAR study was **7** (MIC_99_ = 2.3 µM).

The lead compound **7** was found not to be cytotoxic but was not progressed to *in vivo* efficacy evaluation. Nevertheless, this scaffold has a different pharmacophore from other DprE1 inhibitors with attractive druglike properties that could potentially be used in more extensive SAR programs to improve on DMPK properties.

### MmpL3

The *Mtb* genome encodes genes for 13 different MmpL (mycobacterial membrane protein, large) family transporters and one of these, MmpL3 is the only protein found to be essential under every *in vitro* and *in vivo* growth condition (Domenech et al., 2005; Melly and Purdy, 2019). MmpL3 plays a key role in mycolic acid transport and regulated down-regulation of expression as well as its inhibition leads to the intracellular accumulation of trehalose monomycolate (TMM) with concomitant depletion of cell wall associated mycolates (Grzegorzewicz et al., 2012; Varela et al., 2012). Many chemically diverse scaffolds including the known anti-tubercular clinical candidate SQ109 have been identified as MmpL3 inhibitors giving this protein the dubious credentials as a promiscuous drug target (Tahlan et al., 2012; Chiarelli et al., 2016; Lee and Pethe, 2018).

#### Adamantyl Ureas

The adamantyl urea scaffold was first identified from a whole-cell HTS of ~12K compounds of a commercially-available library (Brown et al., 2011). The structural similarity of the hit with inhibitors of the human soluble epoxide hydrolase (sEH), an enzyme involved in detoxification of reactive epoxides, suggested that the hit may inhibit *Mtb* epoxide hydrolases despite the individual non-essentiality of these (EphB and EphE) targets (Brown et al., 2011). The initial SAR program to evaluate properties monitored whole-cell activity along with inhibition of EphB and EphE as well as sEH and demonstrated that the urea linker (B) was essential for *Mtb* activity. The secondary carbon-linked adamantyl phenyl urea **8** (MIC_90_ = 0.01 µg/mL) was 40-fold more active than the tertiary carbon-linked adamantyl (MIC_90_ = 0.4 µg/mL) in A. Furthermore, poor correlation was observed between whole-cell activity and bacterial epoxide hydrolase inhibition. Overall, the urea derivative with one cycloalkyl (adamantane or cyclooctane) and one substituted phenyl showed excellent *Mtb* potency (MIC_90_ = 0.01 μg/mL) with low cytotoxicity, but suffered from poor solubility, high human plasma protein binding, inhibition of the human soluble epoxide hydrolase the consequence of which remains unpredictable clinically as well as poor bioavailability.

The target of the adamantyl urea hit was confirmed to be MmpL3 by a combination of resistance mutation mapping and demonstration that compound treatment resulted in TMM accumulation inside cells with concomitant reductions in cell wall associated mycolates (Grzegorzewicz et al., 2012). Additional studies have shown that genetic down-regulation of *mmpL3* expression results in enhanced susceptibility to inhibitors such as the adamantyl ureas (Li et al., 2016).

In a follow-up study, a library of 1,600 adamantyl ureas was screened for whole-cell activity to identify additional leads to extend the SAR study (Scherman et al., 2012). This showed that the polarity of the adamantyl-phenyl-ureas could be improved somewhat by hydroxylation and carboxymethylation of the phenyl group (C) without loss of *Mtb* potency. However, lipophilicity of these compounds remained high and none of those tested showed evidence of *in vivo* efficacy. To reduce lipophilicity, the phenyl group was replaced with different heteroaryls and it turned out that isoxazole, thiazole, oxadiazole and pyrazole were tolerated thereby increasing solubility (North et al., 2013). Compound **9** (MIC_90_ = 0.10 µg/mL) was less potent than **8** but had improved solubility, selectivity over sEH and lower cytotoxicity. Poor solubility and potent inhibition of sEH, which has unknown consequences, has impeded the further progression of the adamantyl ureas.

#### Indolecarboxamides

The antitubercular activity of the indolecarboxamide core was identified by two independent groups. The Kozikowski group identified compound **10** (**Table 1**) (MIC_90_ = 0.93 µM) from a phenotypic HTS of a library of ~7K compounds against *Mtb*. It was found to be cidal against *Mtb* with an excellent selectivity index (Onajole et al., 2013) and resistant mutants mapped to *mmpL3* (Lun et al., 2013). To improve antitubercular activity, about 40 analogs were tested which showed that the indole (A) could not be replaced with other heterobiaryl rings. In addition, both methyl substituents were necessary although they could be replaced with fluorine (or trifluoromethyl) with notable improvements in whole cell activity. The methylation of the -NH of the indole or the amide linker resulted in loss of activity, suggesting that both hydrogens interact with the protein binding site. The cyclohexyl ring (B) could not be replaced with aryl, heteroaryl, or heterocycles although a hydrophobic cycloalkyl improved potency with the cyclooctane analog the most potent (MIC_90_ = 0.013 µM) (Onajole et al., 2013).

Researchers at Novartis identified the same hit **10** from cell-based phenotypic HTS of a 2 million compound library (Kondreddi et al., 2013; Rao et al., 2013) and showed that MmpL3 was the target based on resistance mutation and analysis of cell wall mycolates in treated cells. Crystal structures of MmpL3 with the indolecarboxamide showed that this compound occupied the same binding site as SQ109 and the adamantyl ureas (Zhang et al., 2019a). The SAR was designed to address low solubility, high lipophilicity, and poor microsomal stability of this series. Similar observations were made with respect to the essentiality of indolecarboxamide core, substituents in part A, the indole and amide linker -NH group, and the lipophilic cycloalkyl in B. To avoid the bulky and hydrophobic multi ring system, different cyclohexyl moieties with different stereoselectivity and substituents were evaluated confirming the relationship between whole-cell activity and lipophilicity of this scaffold. The most advanced molecules including **12** (MIC_90_ = 0.015 µM) showed promising PK properties as well as *in vivo* efficacy in an acute mouse model (Kondreddi et al., 2013; Rao et al., 2013).

Based on the SAR of the above studies, 40 more analogs, mostly modified in the cyclohexyl (B) were evaluated. This led to the identification of **11** (MIC_90_ = 0.012 µM) which, despite similar activity and lipophilicity to **12**, had improved *in vivo* PK properties and a better dose-dependence during *in vivo* efficacy evaluation compared to **12**. This indolecarboxamide is currently being progressed in the drug development pipeline (Lun et al., 2019).

#### Tetrahydropyrazolo pyrimidine carboxamides

In 2013, the tetrahydropyrazolo pyrimidine carboxamide series was identified as a whole-cell active in several independent screens of diverse small-molecule collections (Maddry et al., 2009; Yokokawa et al., 2013). The stereochemistry of **13** (**Table 1**), identified from a Novartis compound collection, was an important clue suggesting a specific target (Remuinan et al., 2013; Yokokawa et al., 2013). Resistance-conferring mutations mapped to the *mmpL3* gene and the expected phenotype of MmpL3 inhibition associated with TMM accumulation was observed (Remuinan et al., 2013). The cidality against *Mtb in vitro* and during growth in macrophages along with an excellent selectivity window in cytotoxicity studies prompted further evaluation.

From the initial SAR study, the key pharmacophore was defined to include the secondary amine of the tetrahydropyrimidine ring, the amide linker, and the pyrazole ring as well as the absolute stereochemistry. However, **13** (**Table 1**) (MIC_90_ = 0.15 μM) suffered from several liabilities including high lipophilicity, high plasma protein binding and low aqueous solubility. To address the high lipophilicity, the phenyl group in A was replaced with 2-pyridyl and 2-furyl or substituted with polar functionalities at the *para* position to reduce cLogP which had a favorable effect on potency. However, none of these modifications improved solubility or decreased plasma protein binding. Interestingly, aqueous solubility increased considerably on replacement of the trifluoromethyl group on the tetrahydropyrazolo pyrimidine with difluoromethyl with an associated drop in plasma protein binding. Attempts to increase solubility by replacement of B with a pyridyl group improved physical properties but was detrimental to whole-cell activity. Four analogs including **14** showed *in vivo* PK properties that warranted further exploration of chemotherapeutic efficacy but only **14** resulted in reduction of bacterial burdens after 4 weeks of treatment at 100 mg/kg of infected mice to values half a log below the bacterial loads at the start of treatment. A similar study of the lead compound from the group at GSK led to a compound with attractive PK properties that showed a 1-log reduction in bacterial burdens in infected mice at 100 mg/kg, superior to that achieved with 30 mg/kg moxifloxacin (Remuinan et al., 2013). The two studies are, however, not comparable since the mice were treated at different stages of infection and with different drug formulations. Despite the promising *in vitro* and *in vivo* efficacy of this scaffold, significant challenges regarding physicochemical and ADMET properties remain with other MmpL3 scaffolds being superior to this series.

#### Spirocycles

The 1-oxa-9-azaspiro[5.5]undecane termed the spirocycle core was a whole-cell active cluster selected from a HTS campaign of GSK’s compound collection (Ballell et al., 2013), represented by compound **15** (**Table 1**). Resistance-conferring mutations mapped to *mmpL3* and MmpL3 inhibition was suggested by TMM accumulation. The compound was cidal to *Mtb in vitro* and during growth in macrophages but suffered from moderate selectivity window as well as low microsomal stability (Remuinan et al., 2013).

To address these challenges, a focused library of analogs was evaluated for SAR based on whole-cell activity (Guardia et al., 2018). Part A was explored by synthesis of 25 derivatives which revealed that aryl group was more favorable than heteroaryls. However, the activity and microsomal stability were sensitive to the substitution pattern of the aryl ring. Lipophilic and bulky substitutions in the benzyl group including the biphenyl ether improved antitubercular activity. Microsomal clearance of the biphenyl ether analogue was partly blocked by substitution at the *para*-position with, for example, a trifluoromethoxy group as in **16**. Modification of the methylene linker between the bicyclic ring (A) and the spiropiperidine (B) with ketone, sulfone, or amide resulted in complete loss of activity. The piperidine ring could not be changed into a 5-membered pyrrolidine or flexible aliphatic secondary amine. Despite the known lability of piperidine rings to metabolic degradation, there was no effort to block this site to increase microsomal stability. Modification of the tetrahydropyran with different oxygen-containing rings did not improve potency or metabolic stability. To overcome potential oxidation of the thiophene, different replacements were evaluated where removal retained some potency with the replacement being benzene although substituents on the benzene ring to block metabolism resulted in loss of activity. The best analog **16** (MIC_90_ = 0.06 µM) had good microsomal stability and acceptable *in vivo* PK properties. *In vivo* efficacy evaluation of **16** showed that doses as low as 11.6 mg/kg could reduce bacterial loads in mice below that at the time of treatment initiation. Nevertheless, **16** suffers from high lipophilicity, effects on eukaryotic cellular health, inhibition of the human ether-a-go-go related gene (hERG) channel, potential for oxidation at the thiophene sulfur and poor solubility. All attempts to solve these issues in the same molecule failed with **16** remaining the front-runner.

### MurI

Glutamate racemase (MurI), essential in *Mtb*, is a pyridoxal phosphate (PLP)-independent amino acid racemase that racemizes L-glu to D-glu, a building block of the MurNAc-linked and MurNGly-linked peptides of peptidoglycan (Fisher, 2008). The established clinical utility of other targets in peptidoglycan biosynthesis makes it an attractive drug target (Fisher, 2008). The *Mtb* MurI may be highly vulnerable to inhibition since cellular D-glu levels are low and the enzyme has poor catalytic activity, suggesting that it functions as a chokepoint in peptidoglycan biosynthesis (Prosser et al., 2016). Recently, the flavonoids naringenin and quercetin were shown to inhibit the *Mtb* glutamate racemase (Pawar et al., 2020). The structural and kinetic characterization of the *Mtb* glutamate racemase (Poen et al., 2016) has been an important contribution to drug discovery programs against this target.

#### Benzoxazoles

A benzoxazole derivative **17** (**Table 1**) was identified as a novel *Mtb* bacterial glutamate racemase inhibitor from screen of 650 diverse compounds, which was based on a thermal shift assay of the purified *Mtb* enzyme in the presence of D-glu (Malapati et al., 2018). The inhibition of MurI by **17** was confirmed against the recombinantly expressed *Bacillus subtilis* (IC_50_ = 20.7 µM) homolog because the WT recombinant *Mtb* protein is known to be inactive *in vitro* (Poen et al., 2016; Malapati et al., 2018).

The SAR exploration entailed the synthesis of 27 compounds which were tested for activity in the *B. subtilis* glutamate racemase assay followed by *Mtb* MIC testing. However, the SAR of the methyl group on the benzoxazole and benzyl group in A were dependent on the linker, urea and thiourea, complicating interpretation based on the limited number of analogs tested. The antitubercular activity of several compounds including **18** (IC_50_ = 1.1 µM, MIC_90_ = 8.7 µM) was also tested in the presence of the efflux pump inhibitors, Verapamil and Piperine, which showed that several had enhanced activity against cells when efflux was inhibited suggesting that several compounds were substrates of such efflux systems although the SAR for efflux was not established.

The potential binding mode of the compounds was explored by *in silico* docking against the *B. subtilis* and *Mtb* glutamate racemase crystal structures which suggested that these bound to an allosteric site. The active compounds were shown to be cidal against replicating as well as non-replicating *Mtb* and shown to have *in vivo* efficacy against *M. marinum* (a related mycobacterial species often used as model for aspects of *Mtb* pathogenesis) in infected zebrafish. Further studies will be critical to demonstrate on-target inhibition in *Mtb* cells given that compounds with no activity in the enzyme assay had whole-cell activity. Furthermore, there is currently no information available regarding the *in vitro* DMPK liabilities of this series.

## Antitubercular Agents Inhibiting Mycolic Acid Biosynthesis

Targets in mycolic acid biosynthesis have already been validated for their chemotherapeutic potential as evidenced by existing drugs inhibiting mycolate biosynthesis (isoniazid, ethionamide, prothionamide, thiacetazone, perchlozone) that are in clinical use or have been used historically in TB drug regimens (Abrahams and Besra, 2018). Mycolic acid biosynthesis occurs by the Type I and Type II fatty acid synthases (FAS) where the FAS I protein synthesizes the α-chain of the mycolates as well as the precursors of the multi-protein FAS II system (KasA, KasB, MabA, HadAB/HadBC, InhA) that synthesizes the longer chain β-hydroxy fatty acid of the mycolate. The FAS II-generated meromycolate is further modified by cyclopropane, methoxy or keto groups by a panel of enzymes, several of which have been proposed as drug targets based on the critical role of these modifications in cell wall permeability, persistence and pathogenesis (Abrahams and Besra, 2018). Acyl-CoA carboxylase (ACC) and FadD32 activate and load the FAS I and FAS II acyl chain, respectively, onto polyketide synthase Pks13 for final condensation to a α-alkyl-β-ketoacyl derivative which is reduced by Rv2509 to generate the final mycolic acid.

### InhA

The pro-drug isoniazid, inhibits the NADH-dependent enoyl-acyl carrier protein (ACP) reductase (InhA), encoded by *inhA*, but its clinical utility is hampered by the high frequency of mutations in activation the catalase-peroxidase (KatG) required for its activation (Bhat et al., 2018) and the wide variation in the human N-acetyltransferases that catalyze drug metabolism (Sim et al., 2014). Identification of InhA inhibitors that do not require activation of the pro-drug and lack variable human metabolism would be of substantial clinical utility of isoniazid while overcoming the problem of high resistance frequencies.

#### Tetrahydrobenzothieno pyrimidines

The tetrahydrobenzothieno pyrimidine **19** (**Table 2**) was identified as a non-cytotoxic inhibitor of *Mtb* growth from a library of 300 compounds that were known inhibitors of the *Plasmodium falciparum* enoyl-acyl carrier protein reductase (Vilcheze et al., 2011). Unlike isoniazid, **19** (MIC_99_ = 1 µM) was cidal against non-replicating *Mtb*, as well as drug-resistant strains including those with *katG* mutations resistant to isoniazid.

**Table 2:** Antitubercular agents inhibiting mycolic acid biosynthesis.

No.	Target	Scaffold	SAR plan from hit	Most advanced analogue	*In vivo* efficacy	Ref.
1.1	InhA	Tetrahydro benzothieno pyrimidines	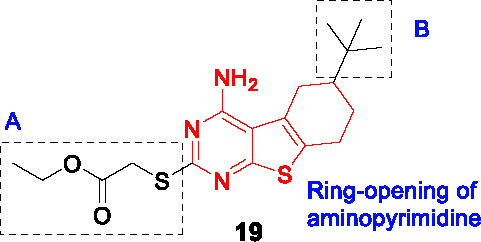	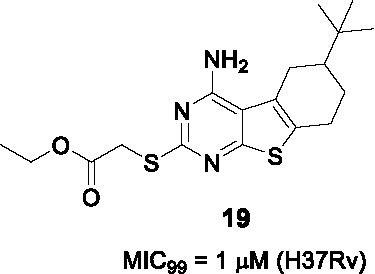	N/D*^a^*	(Vilcheze et al., 2011)
1.2		L-Prolinamides	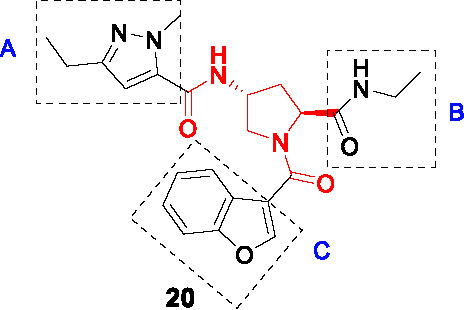	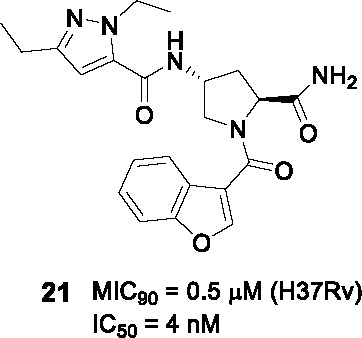	No*^b^*	(Encinas et al., 2014)
1.3		Thiadiazolyl methylthiazoles	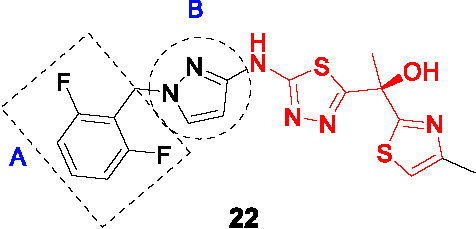	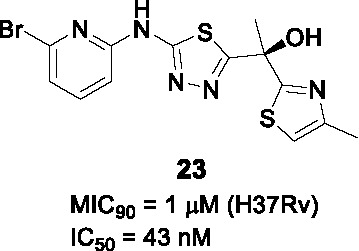	Yes*^c^*	(Sink et al., 2015)
1.4		4-Hydroxy-2-pyridones	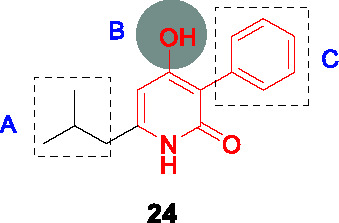	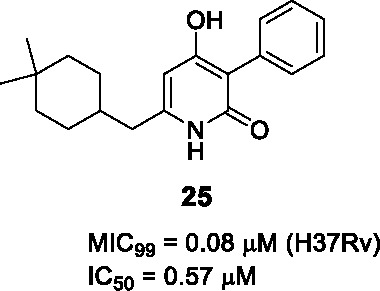	Yes	(Manjunatha et al., 2015; Ng et al., 2015)
1.5		Tetrahydropyranyl methylbenzamides	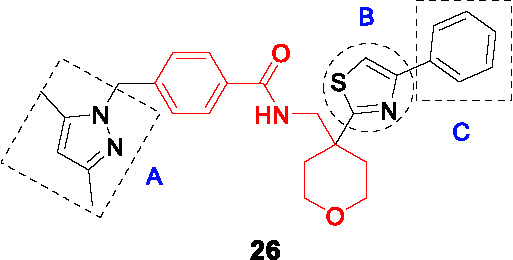	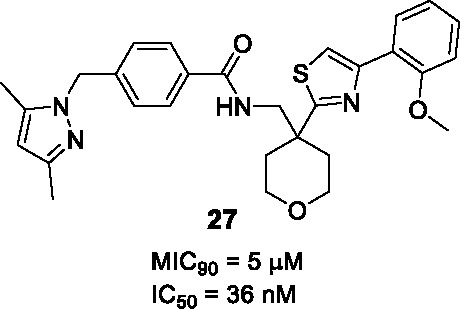	N/D	(Pajk et al., 2016)
2.1	KasA	Indazole sulfonamides	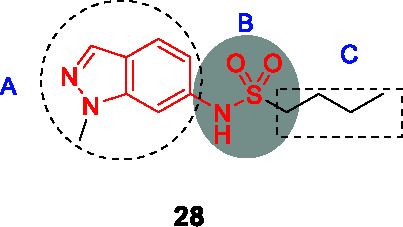	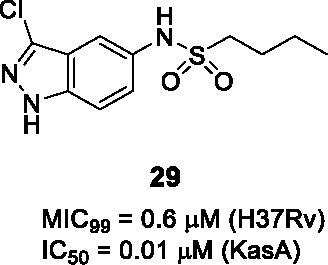	Yes	(Kumar et al., 2018; Cunningham et al., 2020)
3.1	Pks13	Benzofurans	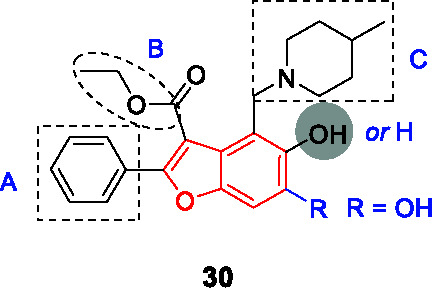	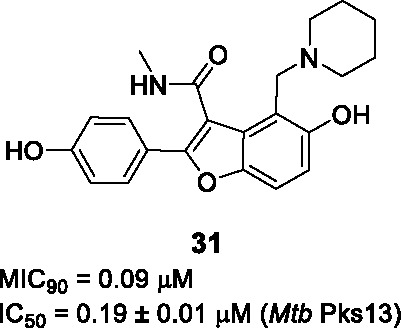	Yes	(Ioerger et al., 2013; Aggarwal et al., 2017)

^a^N/D, not determined; ^b^No, inactive in an acute TB infection mouse model; ^c^Yes, active in an acute TB infection mouse model.

Only 6 analogs of **19** were employed to explore the SAR against *Mtb* cells. In part A, the thioacetate group was essential with only methyl ester being active where the amide, alcohol, or removal of the ethyl ester resulted in complete loss of activity. However, this set of compounds provides limited insight into overcoming the metabolically labile ester functionality. The deletion of *t-*butyl in B was detrimental to potency. Ring-opening of aminopyrimidine resulted in loss of activity although data interpretation is complicated by the fact that the reported compound had simultaneous replacement of the thioacetate with a benzamide.

The on-target effect of **19** was confirmed by demonstrating that it resulted in inhibition of mycolate biosynthesis in *Mtb* with additional depletion of FAS I products suggesting effects on both FAS systems. This notion was confirmed by the lack of resistance conferred by InhA over-expression as well as inability to generate resistant mutants. Extended SAR studies are critical for development of **19** in order to address the ester liability as well as to understand and improve aspects important for *Mtb* activity, cytotoxicity and solubility.

#### L-Prolinamides

A DNA-encoded library (DEL) where large small-molecule libraries containing short DNA fragments as identification bar codes was screened against InhA in the absence or presence of NAD^+^ or NADH (Encinas et al., 2014). An aminoproline hit from the initial screen was used to design an additional DEL library of 16.1 million compounds based on 22 orthogonally protected diamino acid scaffolds with over 800 amine-capping building blocks. Analysis of the next round of hits confirmed the stereoselective 4-aminoproline core with a pyrazole amide moiety as essential to InhA binding. From the analysis, a non-DNA-tagged compound **20** (**Table 2**) was synthesized and confirmed as the best hit against InhA (IC_50_ = 34 nM). Based on X-ray crystallographic analysis and enzyme inhibition data, the interaction of the 4-aminoproline central core with InhA and its chirality were established to be essential. Moderate intrinsic clearance of **20** in a hepatocyte assay prompted metabolite identification studies showing *N*-dealkylation of the ethylamide (B) and the pyrazole (A) (**Table 2**) as well as oxidation products.

The SAR study of **20** was performed to improve enzymatic activity and antitubercular potency while decreasing metabolic liabilities. The *N*-alkyl substituted pyrazole ring (A) was essential for activity with the diethylpyrazole favored. The pyrazole could not be replaced with other heteroaryls, while polar substituents or bulky aromatic substituents on the pyrazole ring increased metabolic stability, these led to significant drops in whole-cell potency. Part B, the site of the DNA-anchor in the DEL, tolerated certain modifications where deletion or replacement of the amide with amine retained InhA inhibition, but the resulting compounds lost whole-cell activity. Replacement of B with methyl glycinate showed excellent activity but, the known lability of esters prevented further consideration. The primary amide of B had the best physicochemical and ADME properties. The benzofuran (C) was critical for enzymatic inhibition where other modifications show no significant improvement. The lead **21** was selected by on InhA and *Mtb* activity (IC_50_ = 4 nM, MIC_90_ = 0.5 µM, intracellular MIC_90_ = 0.24 µM), and acceptable PK properties. However, it lacked *in vivo* efficacy in a murine acute infection model. Additional studies are required to understand the disconnect between *in vitro* and *in vivo* activity.

#### Thiadiazolyl methylthiazoles

The potent, tetracyclic, thiadiazole-based inhibitor of InhA, **22** (**Table 2**), was discovered in GSK’s HTS campaign to discover novel InhA inhibitors, and further biologically validated and co-crystallized with InhA by AstraZeneca revealing the binding mode in the active site (Shirude et al., 2013). Subsequent work aimed to improve physicochemical properties and drug-likeness of **22** (IC_50_ = 4 nM, MIC_90_ = 0.2 µM) (Sink et al., 2015). The thiadiazole and the -NH linker between the pyrazole and thiadiazole rings made hydrogen bonds with the InhA active site and were retained as was the right-hand side thiazole which forms hydrogen bonds with the ribose of the NAD^+^ cofactor. SAR focused on modifying the benzyl (A) and the pyrazole (B) since these made less contacts with InhA. The stereospecific (*S*)-hydroxyl group was the eutomer−the pharmacologically active enantiomer−of the drug although only the racemates were tested for the SAR.

In a focused library of 23 tricyclic analogs, removal of the A ring to reduce lipophilicity was not tolerated without changing the pyrazole (B). Replacement of the pyrazole with different aryl or heteroaryl rings showed that the 2-pyridine retained moderate enzymatic and *Mtb* inhibition with this substitution allowing deletion of the phenyl (A). Hydrophobic and electron withdrawing groups in the α-position of this pyridine, increased potency with the racemic **23** containing a 2-bromopyridine selected as most promising. Chiral separation to yield (*S*)-**23** confirmed this as the most advanced compound. It should be noted that 2-bromopyridines are generally avoided in medicinal chemistry due to reactivity and potential toxicity. Surprisingly, the solubility of tricyclic **23** was not better than the tetracyclic **22**. Nevertheless, **23** showed promising PK properties, good permeability, no hERG signal, and an acceptable CYP inhibition profile. Although *in vivo* evaluation of **23** in infected mice showed limited efficacy, this study was an excellent example of minimizing structural complexity based on a co-crystal structure.

#### 4-Hydroxy-2-pyridones

Novartis identified a new class of 4-hydroxy-2-pyridone derivatives as direct InhA inhibitors, through whole-cell phenotypic HTS of over 2 million compounds against *Mtb* (Manjunatha et al., 2015; Ng et al., 2015). The hit **24** (**Table 2**) was selected based on its drug-like properties, low cytotoxicity, and novelty with promising PK properties and potency (MIC_99_ = 2.5 μM).

The SAR study explored 38 analogs with structural modifications (Ng et al., 2015). Changing the core to methoxy pyridine, pyranone, or quinolinone as well as substitution at the pyridone NH reduced potency. Different replacements of the phenolic OH in B were not tolerated, suggesting the essentiality of the hydroxypyridone core. Replacement of the isopropyl (A) with more lipophilic moieties like dimethylcyclohexyl improved potency. For part C, loss of activity was observed when the phenyl ring was changed to cyclohexyl, pyridyl, or benzyl. Compound **25** (MIC_99_ = 0.08 µM) had the best antitubercular activity albeit with some loss of solubility due to increased lipophilicity.

The target was demonstrated to be InhA by analysis of resistant mutants, InhA over-expression, InhA binding assays, co-crystallography and analysis of newly synthesized fatty acids and mycolates. Importantly, as for other direct InhA inhibitors, clinical strains with *katG* mutations remained sensitive although those with *inhA* promoter mutations were more resistant. Promising *in vitro* ADMET and *in vivo* PK profiles prompted *in vivo* efficacy studies of **25** in the acute mouse model showing comparable efficacy to ethambutol, both at 100 mg/kg, highlighting the drug development potential of this series.

#### Tetrahydropyranyl methylbenzamides

This scaffold exemplified by **26** (**Table 2**) was identified by GSK in an HTS campaign against InhA with good enzyme inhibition and modest MIC (IC_50_ = 20 nM, MIC_90 =_ 11.7 μM). It showed moderate cross-resistance against isoniazid-resistant strains with an *inhA* promoter mutation (Pajk et al., 2016). Co-crystallization of **26** with InhA-NAD^+^ revealed similar binding to that of thiadiazole inhibitor **22** although this information did not guide SAR studies.

To explore the SAR, 18 analogs of **26**, modified in the pyrazole (A), thiazole (B), and phenyl (C) were made while conserving the tetrahydropyranyl methylbenzamide core. Introduction of different heteroaryl or heterocyclic rings instead of the pyrazole, linker modification instead of the methylene between the pyrazole and phenyl, did not improve activity confirming their protein interactions based on the crystal structural. The right-hand side B and C were more accessible for modifications as long as the two aromatic rings were juxtaposed. Compound **27** was selected as the most advanced molecule (IC_50_ = 36 nM, MIC_90_ = 5 µM) with an acceptable selectivity index. The superiority of this scaffold above the many other InhA inhibitors remains to be established.

### KasA

The mycobacterial β-ketoacyl ACP synthase I KasA, an essential component of the FAS II system, is inhibited by the natural products thiolactomycin (TLM) (Schiebel et al., 2013), platensimycin (Brown et al., 2009), and cerulenin (Parrish et al., 1999) although these inhibitors also target the KasB and FabH β-ketoacyl ACP synthases. In addition, these natural products have poor *Mtb* whole-cell activity or in the case of cerulenin, eukaryotic toxicity with limited progress achieved in synthesizing promising leads for drug development. KasA inhibitors would likely offer the clinical utility of isoniazid but would additionally be predicted to synergize with other FAS II inhibitors.

#### Indazole sulfonamides

The indazole sulfonamide series exemplified by **28** (**Table 2**) was identified in screens of a GSK compound library (Ballell et al., 2013; Kumar et al., 2018). This scaffold possessed the attributes of an attractive compound for drug discovery as evidenced by its *Mtb* potency, selectivity for *Mtb*, excellent physicochemical characteristics and promising *in vitro* DMPK profile (Abrahams et al., 2016). The PK profile of **28** was sufficient to allow *in vivo* efficacy testing which showed reduction in bacterial burdens in both the acute and chronic stages of infection in the mouse model (Abrahams et al., 2016). The target was confirmed as KasA based on a combination of resistant mutant mapping, target over-expression, demonstration that mycolic acid biosynthesis was inhibited in treated cells, and enzyme inhibition. Co-crystallization showed that **28** binds in the channel which houses the meromycolate chain locking the enzyme in an open active site conformation (Abrahams et al., 2016).

In a parallel effort, the same hit (**28**) from the GSK whole-cell active library was identified by D. Alland and coworkers as a potent inducer of the *iniBAC* operon suggesting that this compound inhibited an aspect of mycolyl-arabinogalactan biosynthesis (Kumar et al., 2018). This group similarly identified resistance conferring mutations in KasA, demonstrated inhibition of mycolate biosynthesis and importantly their co-crystallization studies revealed the true binding mode of the compound to its target. The crystal structure revealed that the KasA homodimer bound 4 molecules of **28** in non-overlapping sites in the acyl channels with the sulfonamides of the two molecules forming hydrogen bonds with each other.

A SAR study was designed to address primary metabolic stability of the compound as evidenced by relatively high clearance rates by microsomes while retaining or improving *Mtb* potency (Abrahams et al., 2016; Kumar et al., 2018; Cunningham et al., 2020). The high metabolic turnover of **28** was ascribed to *N*-demethylation of indazole rendering an inactive compound (Kumar et al., 2018). However, a different study revealed that the demethylated analog was equipotent against *Mtb* (*vide infra*). In the SAR study by Kumar et al., a series of *N*-substituted indazoles (A) were synthesized but increasing the length of this substitution led to a drastic drop in *Mtb* potency. Sulfonamide replacement with other functional groups in B resulted in loss of activity with only the thiourea retaining modest activity. Elongation of the *n*-butyl (C) to an alkyl chain longer than *n*-pentyl or with bulkier groups resulted in loss of activity. Repositioning of the sulfonamide group from C-6 to C-5 led to a compound that bound as a single molecule per monomer with somewhat improved whole-cell activity. This SAR study did not yield any advanced molecules with markedly improved metabolic stability.

GSK started their own lead optimization program based on the same indazole sulfonamide hit and its demethylated derivative was found to be equipotent to the initial hit (Cunningham et al., 2020). This SAR study similarly found that the *n*-butyl group (C) was the favored moiety. Progression of the indazole sulfonamide was, however, hindered by its embedded aniline moiety. The indazole amine was confirmed to be mutagenic and its formation *in vivo* was demonstrated by metabolite identification in treated rat urine (Cunningham et al., 2020). To overcome this mutagenic potential, linkers to replace the nitrogen of the sulfonamide were generated but all were inactive. The electronic environment of the sulfonamide linker was modified by various substitutions of the 6-membered ring of the indazole to generate compounds that would not readily be metabolized to the amine derivative, but all analogs were inactive. Modifications to the 5-membered ring of the indazole were tolerated where methyl repositioning from *N*-1 to C-3 of the indazole generated the most active of these analogs with methyl or halogen substitutions showing equipotent whole-cell activity to the initial hit (**29**, MIC_99_ = 0.6 µM). There was, however, not always a correlation between whole-cell activity and *in vitro* enzyme inhibition where certain substitutions led to loss of *Mtb* activity while retaining enzyme inhibitory potential. Co-crystallization studies to understand SAR based on enzyme activity did not fully explain on-target SAR. Despite attempts to reduce mutagenic potential, all active indazole and their derivative cores were shown to contain an embedded amine that was mutagenic hindering further progression. An important conclusion from this study was that phenotypic screening to drive SAR was the best approach to optimize the lead since unknown barriers in either compound uptake, efflux or metabolism yielded inactive compounds despite enzyme inhibition.

### Pks13

The *Mtb* Pks13 is the essential polyketide synthase that catalyzes the last Claisen-type condensation of the FAS I-generated C_26_ α-alkyl branch and FAS II-generated C_40-60_ meromycolate precursors (Portevin et al., 2004). This activity is executed by the concerted activity of its 5 domains which consist of two acyl carrier protein domains, a β-ketoacyl-synthase domain, an acyltransferase domain, and a thioesterase domain. This polyketide synthase activity is unique to bacteria that synthesize mycolic acids and as a result, the prediction was that selective inhibitors that do not affect the host microbiome can be discovered. A thiophene-based scaffold containing a reactive pentafluorophenyl that irreversibly targets Pks13 was shown to be cidal *in vitro* and *ex vivo* suggesting the validity of this target (Wilson et al., 2013).

#### Benzofurans

The benzofuran scaffold represented by **30** (**Table 2**) was identified in a phenotypic screen of a commercial library against *Mtb* and resistance-conferring mutations were confirmed to map to *pks13* (Ioerger et al., 2013). Although the initial hit had good *Mtb* potency, it could not be used for *in vivo* evaluation of drug efficacy since it had a labile ethyl ester and the compound had unacceptably low metabolic stability driven by hydroxylation of the phenyl group (Aggarwal et al., 2017). Aggarwal and co-workers demonstrated that the benzofuran inhibited Pks13 catalytic activity (IC_50_ = 0.26 μM) and co-crystallized it with the thioesterase domain showing that **30** bound to the acyl channel leading to the active site (Aggarwal et al., 2017). This crystal structure enabled a structure-guided drug discovery program to optimize **30** in a manner that maintained or improved enzyme inhibition while getting rid of the labile ethyl ester and modifying the phenyl to decrease microsomal metabolism. An important finding from the structural data was that the *p*-phenol derivative (A) that forms during microsomal metabolism would still bind to the thioesterase domain, a finding confirmed by enzyme and *Mtb* inhibition studies showing that this was not a liability. The ester liability (B) was removed by replacement with a methyl amide which retained enzyme inhibition and whole-cell activity. The methyl piperidine could not be replaced with other groups, but removal of the methyl was favored. The structure-guided efforts did not improve potency against the enzyme but proved to be important in demonstrating that the metabolic “liability” could simply be embraced by generating a microsomal metabolite that was similarly active. *Mtb* potency correlated reasonably well with enzyme activity. The SAR study of **30** (MIC_90_ = 2.3 µM, IC_50_ = 0.26 µM) led to the most active compound, **31** (MIC_90_ = 0.09 µM, IC_50_ = 0.19 µM). This compound had favorable ADMET characteristics with plasma serum concentrations achieved above the MIC value. Compound **31** was efficacious in both the acute and chronic mouse models of *Mtb* infection. While **31** showed no evidence of off-target activity against a panel of relevant human protein targets and no inhibition of major CYP isoforms, it showed inhibition in the hERG assay which is predictive of cardiovascular toxicity and contains a Mannich base as substructure. Future drug discovery efforts on this scaffold would need to address the hERG liability.

Natural product-inspired scaffold hopping based on the benzofuran hit **30** to increase metabolic stability and bioavailability, led to the disclosure of tetracyclic coumestans targeting Pks13 with promising ADME-toxicity data (Zhang et al., 2018; Zhang et al., 2019b).

## Antitubercular Agents Regulating Mycobacterial Respiratory System

Emerging evidence of the clinical utility of bedaquiline (Li et al., 2019), a drug that kills both actively replicating as well as non-replicating *Mtb* (Rao et al., 2008), has invigorated studies aimed at identifying inhibitors of enzymes that fuel the protonmotive force that drives ATP synthase. The protonmotive force is generated by components of the respiratory chain (RC), which consists of at least nine dehydrogenases that feed electrons to the two terminal oxidases or three terminal reductases of aerobic and anaerobic respiration, respectively, *via* the membrane soluble menaquinones (Cook et al., 2014). The environment-dependent plasticity of the RC has raised concerns about the validity of targets in this system (Beites et al., 2019). However, the *in vivo* efficacy of inhibitors of apparently redundant targets in this pathway (Kang et al., 2014; Beites et al., 2019) demonstrates that genetic (in)validation of targets requires further chemical validation to predict the ultimate clinical utility of interfering with these proteins.

### NDH-2

*Mtb* encodes a non-essential type-1 NADH dehydrogenase and two type-2 NADH dehydrogenases which share 67% sequence identity. The type-2 NADH dehydrogenases lack a human homolog and although individually non-essential, *Mtb* was predicted to require at least one functional homolog for viability (Vilcheze et al., 2018). The demonstration that both type-2 NADH dehydrogenases could be deleted in the absence of free fatty acids in the medium, suggested that inhibitors of these dehydrogenases would show condition-dependent activity (Beites et al., 2019). The *in vivo* as well apparent clinical efficacy of phenothiazines, which were thought to inhibit both isotypes NDH-2 and NDH-2A, provided preliminary evidence for the tractability of this target although the potent CNS activity limits the clinical utility of the scaffold (Shirude et al., 2012; Alsaad et al., 2014). It is worth noting that phenothiazines likely have other targets in *Mtb* since a mutant lacking both type-2 enzymes remained equally susceptible to these compounds (Beites et al., 2019).

#### Quinolinyl pyrimidines

The quinolinyl pyrimidine series represented by structure **32** (**Table 3**) was one of five clusters identified in a *Mtb* NDH-2 enzyme-based HTS of 100K compounds from the AstraZeneca corporate collection (Shirude et al., 2012). This scaffold was selected based on drug-likeness, potency against the enzyme and whole-cell activity.

**Table 3:** Antitubercular agents inhibition the mycobacterial respiratory system.

No.	Target	Scaffold	SAR plan from hit	Most advanced analogue	*In vivo* efficacy	Ref.
1.1	NDH-2	Quinolinyl pyrimidines	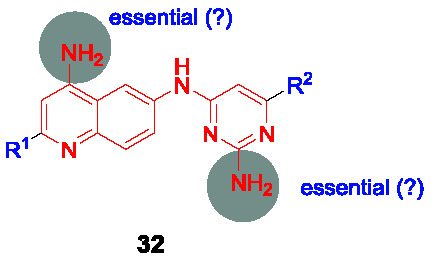	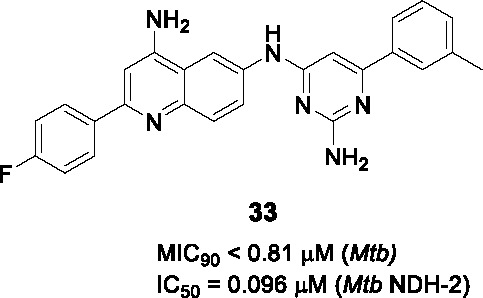	N/D*^a^*	(Shirude et al., 2012)
1.2		Quinolones	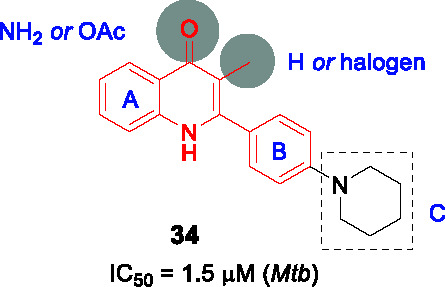	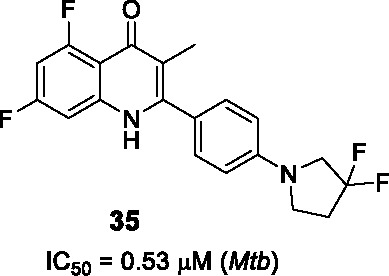	N/D	(Hong et al., 2017)
1.3		2−Mercapto quinazolinones	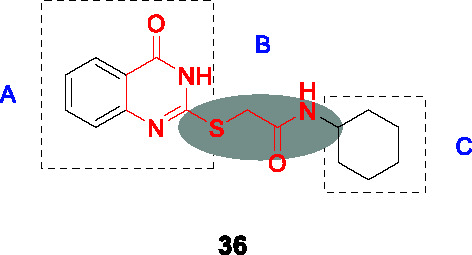	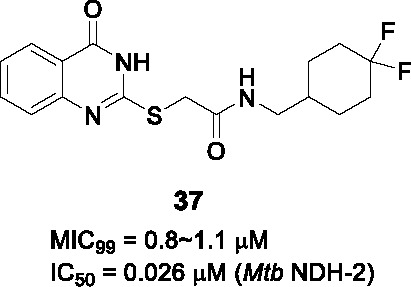	N/D	(Murugesan et al., 2018)
1.4			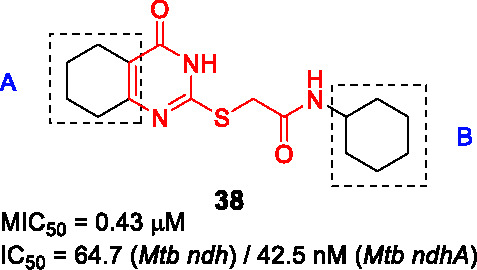	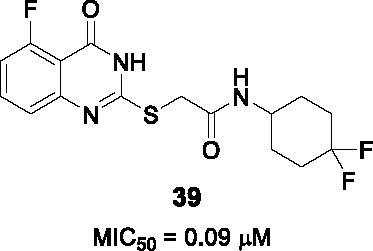	N/D	(Harbut et al., 2018)
2.1	QcrB	Alkyl benzimidazoles	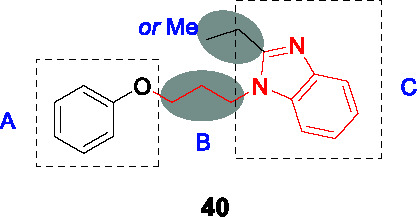	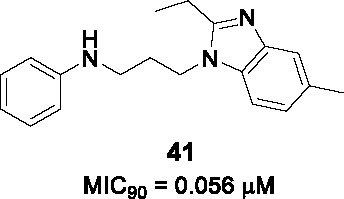	N/D	(Chandrasekera et al., 2015; Chandrasekera et al., 2017)
2.2		Morpholino thiophenes	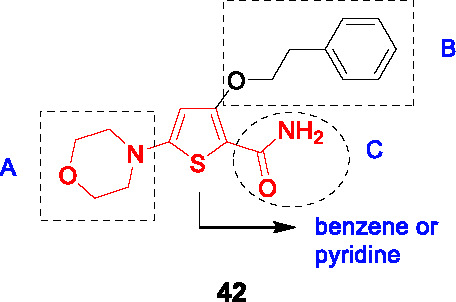	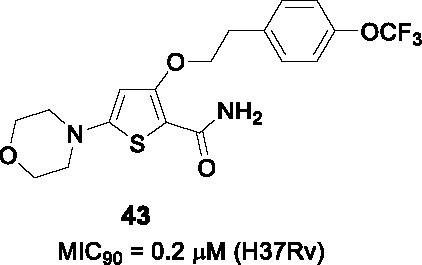	Yes*^b^*	(Cleghorn et al., 2018)
3.1	MenG	Biphenyl amides	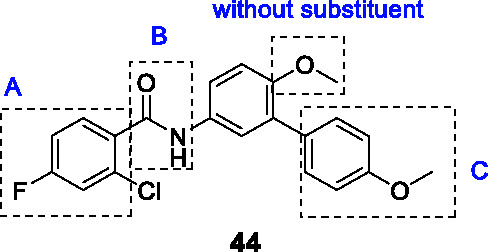	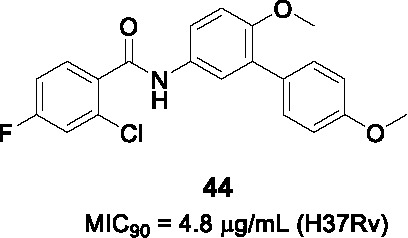	N/D	(Sukheja et al., 2017)

^a^N/D, not determined; ^b^Yes, active in an acute TB infection mouse model.

A small library of 18 analogs was prepared to explore SAR and improve potency against NDH-2. The primary amines on the quinoline and on the pyrimidine moieties were claimed to be critical although the sets of molecules that were reported do not allow pairwise comparisons to support this conclusion. No analogs were reported that individually evaluated the contributions of the quinoline and the pyrimidine. Nevertheless, some compounds like **33** (IC_50_ = 0.096 µM, MIC_90_ < 0.81 µM) showed good enzymatic activities and whole-cell potencies with good correlation between NDH-2 IC_50_ value and *Mtb* MIC. Five selected compounds including **33** were evaluated for *in vitro* PK properties which showed promising results, however, more detailed SAR studies are needed to fully understand the potential of this scaffold.

#### Quinolones

In a different study to identify NDH-2 inhibitors, 11K compounds selected through chemoinformatic methods based on the structures of phenothiazines and 50 quinolones known to target the *Plasmodium falciparum* NDH-2 and cytochrome bc_1_ enzymes were screened against the *Mtb* NDH-2 enzyme (Hong et al., 2017). Compound **34** (**Table 3**) was selected as a starting point based on its whole-cell activity (*Mtb* IC_50_ = 1.5 µM) and predicted DMPK properties.

The SAR of this scaffold was entirely based on whole-cell activity. MIC testing on a quinolone collection of ~350 compounds confirmed that quinolone core was essential and that the phenyl ring in B could not be replaced by heteroaryl or cycloalkyl groups. A focused library of 64 quinolones showed that the most favorable substitution on the A ring was 5,7-difluoro. The quinolone methyl group was important but could be replaced with halogens although this change generated unwanted reactivity. Modification of C ring showed that only amine-containing cycloalkyls with small substituents were favorable in this position. Although the best active analogs including **35** (*Mtb* IC_50_ = 0.53 µM) showed favorable selective indices based on low HepG2 cytotoxicity, most had low microsomal stability due to the metabolic lability of 5- or 6-membered amine-containing heterocyclic moieties in C where single substitution with fluoro or methyl group in the ring did not rescue this liability. Only the gem-difluoro pyrrolidine (**35**) was sufficiently stable to progress to *in vivo* PK which showed poor exposure. Conversion to the acetate pro-drug of the quinolone carbonyl showed better exposure but metID in rat urine indicated extensive biotransformation with a fraction being the required active metabolite. Despite the potent *Mtb* activity of **35** against drug-sensitive and drug-resistant strains, its on-target NDH-2 activity has not been demonstrated. Further optimization is required to address solubility and PK challenges.

#### 2−Mercapto quinazolinones

The 2-mercapto quinazolinones were identified as novel mycobacterial NDH-2 inhibitors by two independent research groups (Harbut et al., 2018; Murugesan et al., 2018).

Murugesan and co-workers identified hit **36** (**Table 3**) from a *Mtb* whole-cell based HTS with the hits selected from its drug-likeness and lack of cytotoxicity (Murugesan et al., 2018). This scaffold had previously been identified in a *Mtb* HTS with mutants mapping to the promoter of the second NDH-2 gene, *ndhA* (Ioerger et al., 2013). The target of 2-mercapto quinazolinones as NDH-2 was confirmed by demonstrating that treatment with compound resulted in depletion of cellular ATP pools, analysis of cellular oxygen consumption indicating inhibition of NADH dehydrogenase activity, showing that resistant mutants mapped to the *ndhA* promoter suggesting compensatory upregulation of the secondary NDH-2 homolog, as well as demonstration of the increased vulnerability of a *Mtb* mutant containing only one copy of the gene encoding NDH-2. Enzyme assays with the recombinant *Mtb* NDH-2 showed that these compounds were a 1000-fold more potent at inhibiting activity than the phenothiazines.

SAR was guided by *Mtb* MIC as well as mouse microsomal stability assays where metabolite identification was used to highlight metabolic liabilities (Murugesan et al., 2018). The pyrimidinone component of the quinazolinone ring (A) was critical for antitubercular activity although the benzene ring could be replaced with cycloalkyl rings. The sulfur of the linker (B) was a liability since it generated adducts with glutathione independent of microsomal activation even though the level of adduct formation for some molecules was low. However, the linker was essential and replacement of sulfur with different atoms, methylation of the amide NH-, or methylation of the methylene resulted in complete loss of activity. Part C was amenable to modification where replacement with different cycloalkyl or methyl-cycloalkyl groups resulted in *Mtb* active compounds. Compound **37** (MIC_99_ = 0.8 µM) was the most advanced molecule in this study based on its improved solubility and microsomal stability (Murugesan et al., 2018).

Harbut and colleagues identified the 2-mercapto quinazolinone scaffold as a hit from a HTS of an 800K compound library in a *Mycobacterium smegmatis* membrane vesicle assay designed to detect inhibitors of NDH-2 by measuring ATP produced by ATP synthase in response to respiratory chain proton pumping after NADH addition (Harbut et al., 2018). The 2-mercapto quinazolinones including **38** were found to inhibit the respiratory chain at the point of NADH dehydrogenase and were selected based on cellular potency (*Mtb* MIC_50_ = 0.43 µM) and lack of cytotoxicity. Similar to the first study, over-expression of the second NDH-2 encoded by *ndhA* conferred resistance. SAR analyses similarly confirmed the essentiality of thioester group in the linker. Further SAR optimization focused on A and B to improve antitubercular activity. Compound **39** (*Mtb* MIC_50_ = 0.09 µM) was the most advanced compound from this study.

In summary, the SAR of both studies was consistent even though a head-to-head comparison of the antitubercular activities is difficult based on different cellular readouts. Nevertheless, the 2-mercapto quinazolinone scaffold has risks that need to be mitigated before it can be progressed based on glutathione adduct formation as well as the finding that the compound had no activity against *Mtb* growing in macrophages possible due to lack of penetration to the phagosome.

### QcrB

QcrB is a subunit of the *Mtb* cytochrome bc_1_ oxidase complex of the RC which re-oxidizes the menaquinols while reducing oxygen with concomitant proton pumping (Cook et al., 2014; Chandrasekera et al., 2017). The finding that QcrB inhibitors are efficacious in murine and non-human primate models of tuberculosis, despite the genetic non-essentiality of this respiratory complex, supported the clinical evaluation of Telacebec (Q203), a QcrB inhibitor, that has completed phase 2 clinical trials with promising results (ClinicalTrials.gov: NCT03563599) (Pethe et al., 2013; Beites et al., 2019; de Jager et al., 2020).

#### Alkyl benzimidazoles

The phenoxy alkyl benzimidazoles represented by **40** (MIC_90_ = 5.2 µM) in **Table 3** was originally discovered as a *Mtb* growth inhibitor in a screen of a 100K commercial library (Ananthan et al., 2009). In the first SAR study, a diverse selection of ~70 analogs was synthesized and evaluated for whole-cell activity (Chandrasekera et al., 2015). This revealed that replacing the phenyl ring in A with other alkyl or heteroaryl groups was generally detrimental although replacement of the phenoxy with a chlorophenyl-oxadiazole-thioxy showed improved activity, but this was not further explored. An electron-donating methyl group on the phenoxy ring (A), especially *m*-Me, improved potency and generated the most potent analog with a *p*-Br substituent. The ether oxygen of the phenoxy could be replaced with sulfur or nitrogen, the latter superior to oxygen. Reducing the length of alkyl linker (B) to less than three carbons was detrimental to activity. The ethyl group in benzimidazole C was critical for the whole-cell activity. The benzimidazole could not be replaced with imidazopyridine but certain substituents like 6-Me on the benzimidazole improved activity 5-fold. The compounds generally suffered from poor microsomal stability and solubility. As with other known QcrB inhibitors, the analogs were found to be bacteriostatic against replicating *Mtb* and bactericidal against nonreplicating *Mtb*.

In a follow-up report, the authors further explored SAR to improve *Mtb* potency and solubility (Chandrasekera et al., 2017). Replacement of the phenoxy with an anilinyl group with diverse substitution patterns served to improve *Mtb* activity and solubility. The chlorophenyl-oxadiazole-thioxy instead of phenoxy was further evaluated resulting in limited improvement. Replacement of the benzimidazole with a simple imidazole resulted in loss of activity. The most potent and least cytotoxic compound from this work was **41** (MIC_90_ = 56 nM, intracellular IC_90_ = 28 nM). The target was determined to be QcrB based on measuring cytosolic ATP depletion and generation of resistant mutants with most active compounds from this work showing cross-resistance to these *qcrB* mutants. The lack of information on *in vitro* ADMET properties for the newly synthesized analogs could imply limited improvement where other more attractive scaffolds warrant more investment.

#### Morpholino thiophenes

A novel morpholino thiophene scaffold was identified from the phenotypic Eli Lilly corporate collection against *Mtb* with the initial hit, **42** (**Table 3**), showing potent *Mtb* activity (MIC_90_ = 0.72 μM) without cytotoxicity (Cleghorn et al., 2018). Preliminary *in vitro* ADMET profiling established good kinetic solubility, moderate CYP inhibition, no hERG liability but high metabolic turnover. The SAR was designed to understand the pharmacophore in parallel with improving microsomal stability.

The thiophene core could be replaced with phenyl or pyridine to improve microsomal stability while maintaining whole-cell activity. Replacement of the morpholine moiety, a known metabolic liability in microsomal stability assays, was not tolerated and protection methods like methylation or bridged approaches did not improve microsomal stability or potency. Modification of the substitution pattern on the phenyl ring (B) improved antitubercular activity and microsomal stability with a *p*-substituent especially favored. Replacement of the phenyl with heteroaryl or alkyl was not tolerated. The ethyl ether linker was also important and shortening its length, replacing oxygen with carbon or constraining the linker by ring formation was detrimental. The primary amide in C could not be removed or replaced with ester, suggesting a role target protein binding. However, the methyl amide or methyl ketone were equipotent but had lower microsomal stability. The most advanced compound **43** showed improved potency (MIC_90_ = 0.2 μM) and microsomal stability, moderate *in vivo* PK profiles, and good permeability but poor thermodynamic solubility driven by the thiophene core. The *in vivo* metabolites of **43** were confirmed to be the oxidized metabolites of the morpholine. Importantly, **43** showed *in vivo* efficacy against *Mtb* in an acute 4-day murine model at 100 mg/kg as evidenced by a small reduction in lung bacterial burdens.

The 2D structural similarity between **43** and Q203 suggested QcrB as the target which was confirmed by cross-screening against a panel of *Mtb* QcrB mutant strains as well as demonstration of dose-dependent cellular ATP depletion.

### MenG

The lipid soluble electron carrier menaquinone is central to the function of the RC under replicating and non-replicating conditions (Cook et al., 2014). Consequently, the essential enzymes involved in biosynthesis of the 1,4-dihydroxy-2-naphthoate core from chorismate, the isoprenoid side chain through the mevalonate pathway as well as final methylation by MenG are considered potential drug targets.

#### Biphenyl amides

The biphenyl amide **44** (**Table 3**) was discovered in a whole-cell screen of *Mtb* designed to detect respiratory inhibitors based on expression of a fluorescent protein driven by a promoter that is upregulated during respiratory inhibition (Sukheja et al., 2017). This non-cytotoxic compound was identified from a set of 168 *Mtb* growth inhibitors with unknown modes of action (MIC_90_ = 4.8 µg/mL). Resistant mutants mapped to *menG* encoding the SAM-dependent methyltransferase that methylates demethylmenaquinone. The inhibition of MenG was confirmed by target over-expression, analysis of newly synthesized menaquinone depletion and rescue by exogenous menaquinones. Compound **44** had excellent bactericidal activity against both replicating and non-replicating cells.

A limited SAR study was performed with 10 analogs to understand the pharmacophore. Briefly, potency was not improved upon halogen removal in the A ring, linker (B) modification by deletion of the carbonyl group or methylation of NH, methoxy replacement with hydroxy or hydrogen, or finally C ring deletion or substitution. Despite the promising novel activity of compound **44** in-depth SAR studies are required for further development.

## Antitubercular Agents Affecting Other Mycobacterial Targets

### Rv0577 (Methyl Glyoxal Detoxification)

#### Pyrimidine imidazoles

The pyrimidine imidazoles were introduced as a new class of antitubercular compounds based on a hit from a chemical library screen against *M. bovis* BCG. The chemical cluster that yielded hit **45** (**Table 4**) was confirmed cidal against *Mtb* (MIC_50_ = 0.65 µM, MBC_90_ = 2.5~5 µM) with an acceptable cytotoxicity profile. A total of 324 analogs were synthesized for lead development, although only 4 of these were published thus preventing an understanding of the SAR and the medicinal chemistry efforts applied to solve key issues. These 4 compounds have the same pyrimidine imidazole scaffold but different modifications in parts A and B. The lead compound (**46**) was potent against *Mtb* (MIC_50_ = 0.036 µM) and PK/PD indices at 100 mg/kg comparable to those of isoniazid at 25 mg/kg. However, **46** and the other 3 active analogs were found to be ineffective in reducing lung bacterial loads *in vivo* as evaluated in the acute mouse model.

**Table 4:** Antitubercular agents inhibiting other mycobacterial targets.

No.	Target	Scaffold	SAR plan from hit	Most advanced analogue	*In vivo* efficacy	Ref.
1.1	Rv0577	Pyrimidine imidazoles	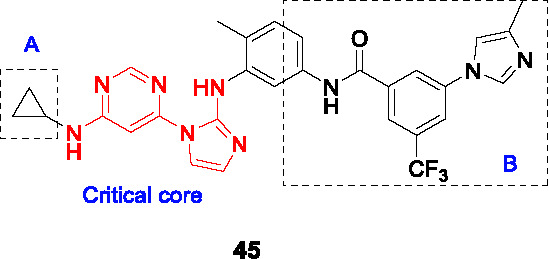	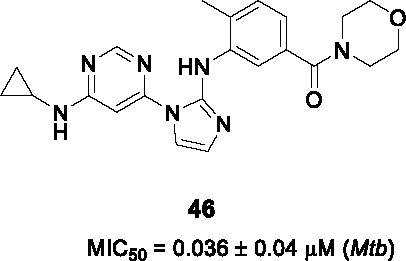	No*^a^*	(Pethe et al., 2010)
2.1	FtsZ	Benzimidazoles	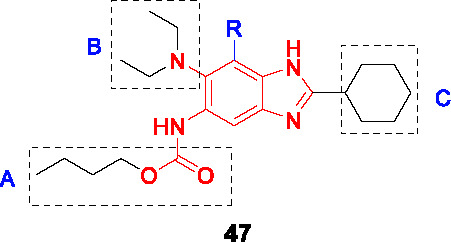	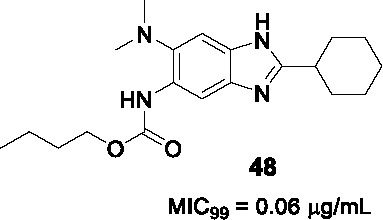	N/D*^b^*	(Kumar et al., 2011; Awasthi et al., 2013)
3.1	EthR	*N*-phenylphenoxy acetamides	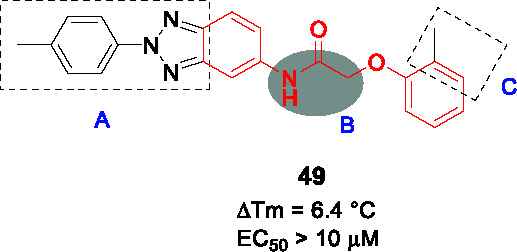	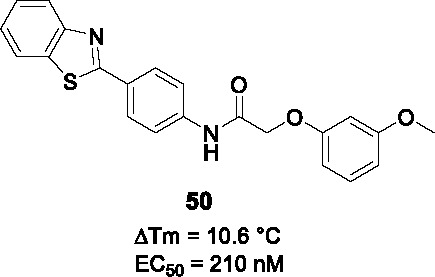	N/D	(Flipo et al., 2012)
4.1	LepB	Arylhydrazones	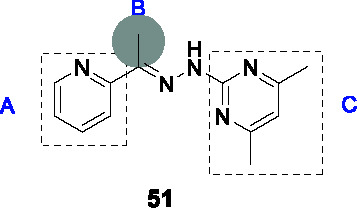	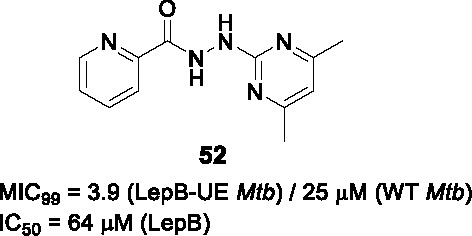	N/D	(Bonnett et al., 2016)
5.1	CysM	Diphenyl ureas	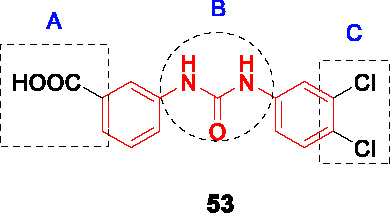	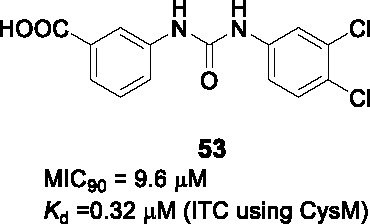	N/D	(Brunner et al., 2016)
6.1	BioA	Benzoyl piperazines	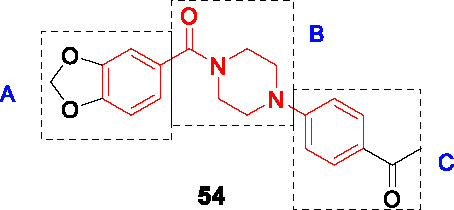	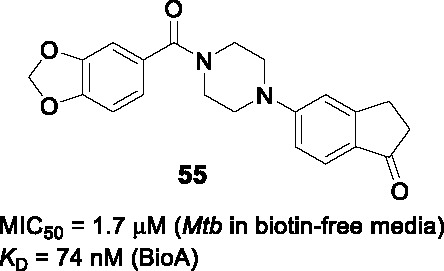	N/D	(Park et al., 2015; Liu et al., 2017)
7.1	IMPDH	Indazole sulfonamides	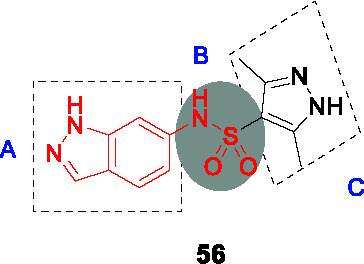	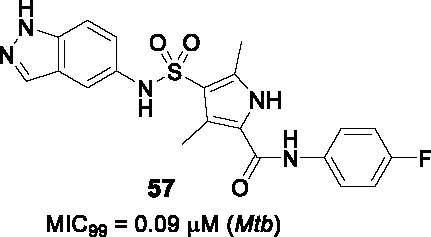	No	(Park et al., 2017)
7.2		Benzoxazoles	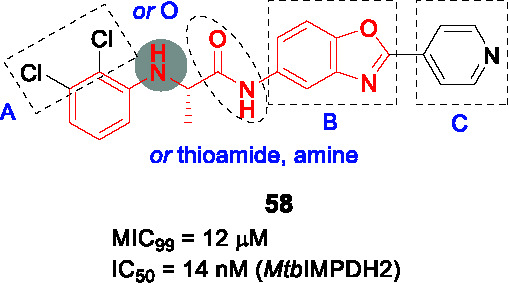	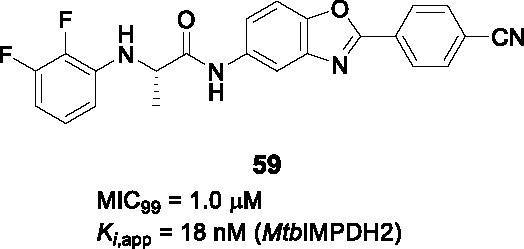	N/D	(Chacko et al., 2018)
8.1	PknA PknB	Pyrazolyl pyrimidinamine	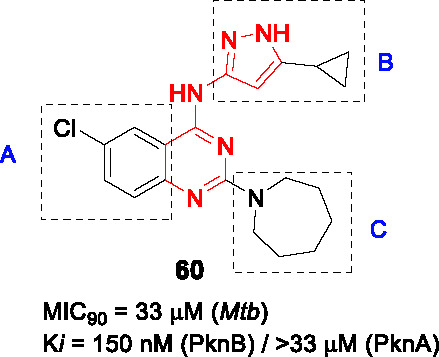	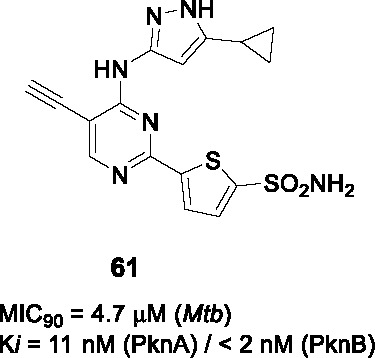	N/D	(Wang et al., 2017)
9.1	CorA	Pyrimidinetrione amides	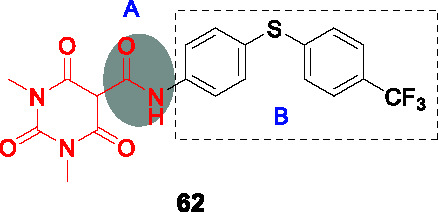	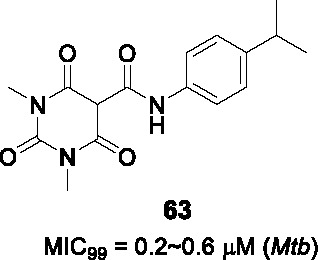	No	(Park et al., 2019)
10.1	DosS DosT	Ureas	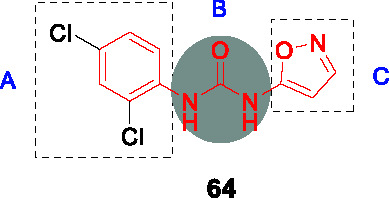	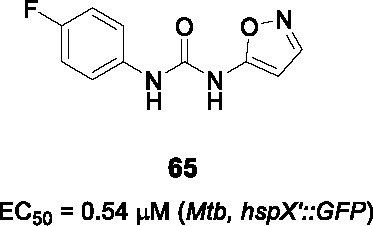	N/D	(Zheng et al., 2020)

^a^No, inactive in an acute TB infection mouse model; ^b^N/D, not determined.

Mechanism of action studies of this pyrimidine imidazole revealed that the whole-cell activity of the analogs was dependent on the presence of glycerol in growth medium where the resistance mechanism was mapped to inactivation of the mycobacterial glycerol kinase resulting in inability to catabolize glycerol as carbon source. Affinity pulldown indicated that the target protein was likely a glyoxylase (Rv0577), a potential enzyme involved in methylglyoxal detoxification which was further corroborated by over-expression studies. This work confirmed that glycerol metabolism is unlikely a vulnerable pathway in the mouse as further evidenced by the lack of phenotype of a *Mtb* glycerol kinase mutant in this mouse model. The dependence of *Mtb* on glycerol metabolism during human infection remains unknown and the over-dependence of TB drug discovery efforts on mouse infection models means this will likely remain so for the future. It also underscores the importance of screening under physiologically relevant conditions *in vitro* to avoid unnecessary and costly drug discovery efforts.

### FtsZ

#### Benzimidazoles

FtsZ is a structural and functional homologue of tubulin, which is a highly conserved and ubiquitous bacterial cell division protein. It polymerizes in the presence of GTP and forms a dynamic helical structure, the Z-ring, at the midpoint of the cell (Errington et al., 2003; Leung et al., 2004). The discovery of FtsZ inhibitors combined with the essentiality of FtsZ in mycobacterial cytokinesis has provided the initiative for further drug discovery efforts against this target (Haranahalli et al., 2016).

The benzimidazole substructure of known tubulin inhibitors like albendazole and thiabendazole (Kumar et al., 2011) served as the starting points for the discovery of new FtsZ inhibitors based on a focused library of 587 trisubstituted benzimidazoles that were screened against *Mtb* yielding compound **47** (**Table 4**) (MIC_99_ = 0.63 µg/mL) without appreciable cytotoxicity (Kumar et al., 2011). The SAR studies around the hit revealed that alkyl carbamates and benzamides had superior activity to alkyl or cycloalkyl amides in A whereas part B tolerated both dialkyl amines and cyclic amines. Many analogs with alkyl amides in R (2,5,7-trisubstitued benzimidazoles) were synthesized but none were as good as 2,5,6-trisubstitued analogs like **47**. The cyclohexyl group in C was favored over aromatic counterparts (Kumar et al., 2011; Awasthi et al., 2013). The lead compound **48**, selected based on its superior potency (MIC_99_ = 0.06 µg/mL), exhibited dose-dependent inhibition of FtsZ polymerization *in vitro* probably by enhancing its GTPase activity. Morphological changes in treated cells were also consistent with FtsZ inhibition.

Knudson and co-workers provided evidence for the *in vivo* efficacy of this series as evidenced by a moderate reduction in organ burdens of treated *Mtb* infected mice (Knudson et al., 2015). However, interpretation of the efficacy data is complicated by the lack of PK data. Further *in vitro* ADMET studies are needed to guide series progression.

### EthR

#### *N*-phenylphenoxy acetamides

Ethionamide is a prodrug that activated by the monooxygenase, EthA, the expression of which is controlled by the EthR transcriptional repressor. Inhibition of EthR enhances the bioactivation of ethionamide hereby potentiating activity of the drug *in vivo* (Willand et al., 2009; Ang et al., 2017). The *N-*phenylphenoxy acetamides were identified as EthR inhibitors with the ability to penetrate the mycobacterial cell wall and membrane in *M. smegmatis* screen of a ~15K library. The assay readout was based on inhibition of binding of EthR to EthA promoter allowing the expression of a measurable enzyme (Flipo et al., 2012). Validated hits were confirmed to bind EthR by fluorescence-based thermal shift assay yielding **49** (**Table 4**) (IC_50_ = 2.9 µM, ΔT^m^ = 6.4 °C).

The X-ray crystal structure of **49** with the EthR showing the *N*-phenylphenoxy acetamide motif as the key pharmacophore for binding, enabled design and combinatorial synthesis of 960 analogs with chemical diversity limited to phenols and anilines introduced at the amide and ether bonds (B). These were screened in the thermal shift assay with hits evaluated for their ability to boost the activity of ethionamide. Briefly, the left-hand side (A) was critical for both binding and growth inhibition because only specific heteroaryls like benzothiazole and *p*-Cl substituted phenyl bound EthR and potentiated ethionamide. For part B, aryl or alkyl substituents on the -NH or α-carbon did not improve binding activity. For the right-hand side (C), the effect of a few substituents on the phenyl ring were tested and although a somewhat more complex SAR, the *m*-OMe or *m*-CN were found to bind EthR as well as boost ethionamide activity. The most active analog **50** was at least 50-fold more active than **49** in boosting ethionamide activity in *Mtb*-infected macrophages (EC_50_ > 10 µM for **49** and 0.21 µM for **50**). Interestingly, the co-crystallization of **50** revealed a different binding mode compared to that of **49**. Expanded SAR studies along with *in vitro* ADMET, PK profiling and *in vivo* evaluation would be needed to advance this scaffold.

### LepB

#### Arylhydrazones

The type I signal peptidase encoded by *lepB* is essential for *Mtb* where it regulates secretion across the cytoplasmic membrane. In an effort to identify inhibitors of this enzyme, a whole cell screen of a 72K commercial library was performed against a *Mtb lepB* hypomorph (Bonnett et al., 2016). The SAR study based on arylhydrazone hit **51** (MIC_99_ = 3.0/18 µM in LepB-hypomorph/WT *Mtb*) showed that part A was sensitive to modification with a pyridine being the preferred functionality. Part B could be replaced by methyl, phenyl groups or even a carbonyl to yield the corresponding picolinohydrazide as in **52** (**Table 4**). Part C also tolerated a larger range of substitutions or different functionalities, with the only requirement being the maintenance of the hydrazone linker. There was no correlation between LepB inhibition in a membrane fraction assay and whole cell inhibition. The compounds were weak inhibitors of WT *Mtb* and for some an on-target effect was suggested by rescue by LepB over-expression. Several compounds were, however, cytotoxic with even the most advanced compound **52** only showing a moderate selectivity window against the LepB hypomorph but not against the WT *Mtb*. Additional SAR analyses with a focus on addressing cytotoxicity, LepB inhibition as well as potential stability issue of arylhydrazones are needed to progress this series.

### CysM

#### Diphenyl Ureas

Low molecular weight thiols such as ergothioneine, mycothiol and γ-glutamylcysteine play critical roles in protecting *Mtb* from oxidative stress with their synthesis requiring cysteine. CysM is one of three pyridoxal phosphate (PLP)-dependent cysteine synthases in *Mtb* the function of which is important under conditions of non-replicating persistence and *in vivo*. A target-based screen of ~17K chemically diverse compounds was performed wherein ligand-binding to the CysM active site resulted in a PLP-dependent fluorescence increase (Brunner et al., 2016). The biphenyl urea **53** (**Table 4**) bound CysM in a concentration dependent manner (*K_d_* = 0.32 µM).

The SAR was guided by co-crystal structures of CysM bound to analogs such as **53** and included 71 compounds. The left-hand side (A) along with its *m*-carboxylate were required for binding as well as enzyme inhibition. Similarly, the urea linker (B) was critical and could not be substituted with thiourea, amide, or sulfonamide. Part C could tolerate substitutions although **53** remained the best analog.

Only a few selected analogs including **53** were evaluated for their antitubercular activities against *Mtb* which showed little correlation with CysM binding although the limited numbers tested allow few conclusions to be made. Compound **53** (MIC_90_ = 9.6 µM) was 4-5-fold less potent against *Mtb* than another analog but showed equivalent cidality to the more potent analog on non-replicating cells suggesting additional off-target effects. The on-target inhibition was validated by rescue with cysteine.

The same set of 71 analogs were subsequently tested for binding and inhibition of CysK1 and CysK2, two alternative cysteine synthases in *Mtb* that differ in sequence, mechanism as well as sulfur substrate from CysM (Brunner et al., 2017). Several showed some binding and inhibition of these enzymes although at least an order of magnitude less potent. Expanded SAR studies, *in vitro* ADMET and PK profiling would be important to perform the much-needed *in vivo* validation of this scaffold.

### BioA

#### Benzoylpiperazines

BioA (7,8-diaminopelargonic acid synthase) is a PLP-dependent aminotransferase required for biotin biosynthesis in *Mtb* (Dey et al., 2010). Its essentiality in *Mtb* at all stages of murine pathogenesis indicated that host biotin is not sufficiently accessible to the pathogen which, along with the ability of known BioA inhibitors to inhibit *Mtb* growth, suggested this as an attractive drug target. Inhibitors against the target were identified in a BioA enzyme-based screen of a ~350K compound library including ~83K diversity-oriented synthesis (DOS) compounds (Park et al., 2015). An extensive panel of counter-screens were performed to ensure lack inhibition of the subsequent enzymatic reaction (BioD), inhibition of general PLP-dependent enzymatic activity as well as of cytotoxicity. Compound **54** (**Table 4**) was one of 16 analogs with a benzoylpiperazine core that had submicromolar affinity for BioA and a moderate MIC_50_ against WT *Mtb* in the absence of biotin which could be rescued by biotin supplementation. The on-target effect was further established by demonstrating enhanced susceptibility in a BioA hypomorph and resistance in an over-expressor strain (0.4 µM in BioA-UE, 31.1 µM in BioA-OE, 5.6 µM in WT-biotin, >50 in WT+biotin). Co-crystallization studies of BioA bound to **54** and analogs allowed mapping of the binding site and the identification of unoccupied cavities around parts A and C, that could be capitalized on for further compound optimization.

Structure-guided compound optimization allowed the design of 34 analogs (Liu et al., 2017) which showed that benzodioxole, 3,4-dichlorophenyl, and *m*-chlorophenyl in A were optimized contacts in the respective binding pocket with corresponding whole-cell activities unlike different heterocycles and alkyls. The piperazinyl carbonyl linker (B) could not be replaced with piperidine or triazole. The acetyl oxygen (C), makes important hydrogen bond interactions to BioA and thus its position was critical for activity. Only inden-1-one bicyclic analog **55** (**Table 4**) had improved BIoA binding and whole-cell activity (0.3 µM in BioA-UE, 2.8 µM in BioA-OE, 1.7 µM in WT-biotin, >50 in WT+biotin) (Liu et al., 2017). Further evaluation with emphasis on *in vitro* ADMET, PK profiling and *in vivo* efficacy evaluation are critical to advance this series.

### IMPDH

Inosine-5′-monophosphate dehydrogenase (IMPDH) catalyzes the NAD^+^-dependent oxidation of inosine 5’-monophosphate (IMP) to xanthosine 5’-monophosphate (XMP) and thus regulates guanine nucleotide pools. It has received considerable attention as a drug target for various pathogens and pathological conditions (Braun-Sand and Peetz, 2010). Although *Mtb* encodes three homologs, only one of these encoded by *guaB2* is essential. As a target for TB treatment, it has emerged as a promising one but still required for more study to validate (Chacko et al., 2018).

#### Indazole sulfonamides

The indazole sulfonamide **56** (**Table 4**), was initially identified from whole-cell based HTS of ~ 100K small-molecule library against *Mtb* (Park et al., 2017). This compound had attractive lead-like properties with lack of cytotoxicity (MIC_99_ = 2 µM). Resistance in mutants was ascribed to promoter mutations as well as amplification of the genomic locus leading to target over-expression. On-target effects were further demonstrated by guanine rescue experiments as well as metabolomic analysis showing effects on nucleotide pools consistent with IMPDH inhibition. The compound was an uncompetitive inhibitor of IMPDH (IC_50_ = 0.8 µM) with structural studies of IMPDH crystals soaked with compound showing extensive interaction with IMP.

The SAR based on a limited number of reported compounds revealed the regioselectivity of the indazole moiety (A) where the sulfonamide linker (B) had a distinct pattern in activity that both the 6- and 5-isomers were but the 7-regioisomer was not (Park et al., 2017). Replacement of the indazole with other heteroaryl groups did not improve activity except for quinoline. The indazole NH- was critical since its methylation resulted in complete loss of activity. The linker sulfonamide (B) was essential with both the sulfonate and reverse sulfonamide being inactive. Part C was more accessible for scaffold optimization where the regioisomer of pyrazole, benzyl, and other heterocycles were inactive although other modifications such as amide substituted pyrroles (**57**) (MIC_99_ = 0.09 µM) improved antitubercular activities. A potential risk of these compounds is generation of the aniline derivative by host metabolism causing potentially mutagenic metabolites.

The series lacked activity in nonreplicating cells and in *in vivo* murine infection model. The latter may have been driven by insufficient compound exposure although host tissue guanine levels were sufficiently high to rescue the IMPDH inhibition. The demonstration that host metabolites can potentially overcome target inhibition is a critical consideration in drug development and particularly relevant to enzymes involved in core metabolism.

#### Benzoxazoles

A benzoxazole series developed to target the less conserved NAD^+^-binding site of the *Cryptosporidium parvum* IMPDH, an enzyme most related to prokaryotic orthologs, was screened for whole-cell activity against *Mtb* with the active compounds further evaluated for inhibition of the *Mtb*IMPDH (Makowska-Grzyska et al., 2015). The co-crystal structure of the stereoselective benzoxazole **58** (*K*_i,app_ = 14 nM, MIC_99_ = 12 µM), one of 37 benzoxazole derivatives, with *Mtb*IMPDH (**Table 4**) revealed the ligand-IMP-*Mtb*IMPDH interactions critical for affinity and selectivity.

Structure-guided optimization of **58** (Chacko et al., 2018) showed that changing the 2,3-dichloro (A) to enhance interaction with IMP and a critical Glu (Glu458) in the binding site increased enzyme inhibition. Modification of the linker by replacement of the aniline with ether was tolerated although replacement of the amide with thioamide or amine decreased whole-cell as well as enzyme inhibition. The benzoxazole (B) which forms important contacts with the protein, could not be replaced it with imidazopyridine. The pyridine in C binds to a hydrophobic part of the pocket that generates selectivity above the human ortholog could be replaced with substituted phenyl, where the *p*-CN substituted phenyl (**59**) yielded a compound with superior enzyme as well as *Mtb* inhibition (*K*_i,app_ = 18 nM, MIC_99_ = 1.0 µM) without serious cytotoxicity. It was confirmed that **59** selectively inhibits bacterial IMPDHs compared to human counterparts.

Surprisingly, the optimized compound **59** showed less evidence of on-target inhibition compared to **58** as demonstrated by guanine rescue although IMPDH hypomorphic and over-expression strains showed the expected enhanced and decreased vulnerability, respectively, to compound inhibition. The inability of exogenous guanine in rescuing *Mtb* from the optimized benzoxazole was interpreted as evidence of target vulnerability in contrast to the conclusion from the indazole sulfonamide series. Microsomal metabolism studies of **59** indicated moderate clearance *in vitro*. PK studies indicated brief exposure above the MIC although high levels of protein binding indicate that the compound needs to be optimized to decrease protein binding while enhancing potency. The major risk in developing IMPDH inhibitors remains the possibility that host guanine can rescue target inhibition although the benzoxazole series may result in a mechanism of inhibition that leads to irreversible disruption of cellular homeostasis.

### PknA and PknB

#### Pyrazolyl pyrimidinamines

The *Mtb* genome encodes 11 serine/threonine protein kinases of which only protein kinase A (PknA) and protein kinase B (PknB) are essential *in vitro* and during growth in host macrophages (Prisic and Husson, 2014). In an effort to identify PknA and PknB dual inhibitors, Wang and colleagues screened a 1,078 compound library based on kinase inhibitor scaffolds against the PknA and PknB kinase domains (Wang et al., 2017). Hits with *K_i_* < 10 µM, were modeled into the PknB crystal structure as well as into the PknA homology model to identify those with optimal binding of both targets to develop dual inhibitors. Quinazoline **60** (**Table 4**) was selected for SAR studies based on its moderate PknB and whole cell *Mtb* inhibition (*K_i_* = 150 nM, MIC_90_ = 33 µM), despite absence of PknA inhibition.

The SAR revealed that the 6-Cl in the benzene (A) could be replaced with other substituents as long as the bicyclic quinazoline was intact. Evaluation of the aminopyrazole (B) showed that none of the extensive panel of aminoheterocycles tested, improved PknB inhibition. Two approaches were used to explore the cyclic amine (C). Firstly, it was modified to primary and secondary amines to retain the sp^3^-conformation but only the cyclobutylamino had better activity. Secondly, it was replaced with different aryls which yielded superior compounds where the sulfonamide substituted aryl group resulted in inhibition of both PknA and PknB. However, whole-cell activity was not improved possibly due to lack of cell penetration or efflux. To address this, they truncated the quinazoline ring (A) into a simple monocyclic pyrimidine ring to reduce lipophilicity, resulting in a potent, dual-targeting pyrazolyl pyrimidinamine **61** (**Table 4**) (*K_i_* = 11 nM for PknA, < 2 nM for PknB, MIC_90_ = 4.7 µM). Although terminal alkynes are generally considered biologically inert, potential oxidative addition by cytochrome P450 enzymes would decrease metabolic stability of **61** (Lehmann et al., 2016), a liability not yet addressed for this analog. To further advance this kinase inhibitor, selectivity above an extended panel of human kinases would need to be demonstrated along with *in vitro* ADMET, PK profiling followed by *in vivo* efficacy studies to provide the proof of concept needed to validate PknA/B as a drug target.

### CorA

#### Pyrimidinetrione amides

The pyrimidinetrione amide **62,** identified from a HTS against *Mtb*, is based on a barbiturate scaffold which has been developed for wide range of biologically active compounds (Park et al., 2019). The SAR study showed that the amide linker (A) was critical for activity because replacement with ketone or sulfonamide resulted in complete loss of activity against *Mtb*. Modification of part B with alkyl, cycloalkyl or heteroaryl did not increase potency. Many analogs had poor selectivity based on cytotoxicity against HepG2 cells. Only *p*-alkyl substituents on the phenyl ring like isopropyl-substituted **63** (MIC_99_ = 0.2~0.6 µM) had improved *Mtb* potency with an excellent selectivity index.

Mutants against **62** mapped to the *corA* gene encoding a Mg^2+^/Co^2+^ transporter. Inhibition of CorA was validated by Mg^2+^-dependent rescue of growth inhibition and *in vitro* binding of the Mg^2+^-**63** chelate to recombinant CorA demonstrated by thermal stability assays. Despite excellent cidality against *in vitro* replicating and non-replicating *Mtb* as well as *Mtb* growing in macrophages, **63** lacked *in vivo* efficacy in mice driven by poor PK at the highest tolerable dose. Further development of the pyrimidinetrione amide scaffold is hampered by cytotoxicity. However, this work demonstrated the critical role of CorA in regulating bacterial Mg^2+^ homeostasis with *in vivo* validation likely requiring discovery of novel CorA-binding scaffolds.

### DosA and DosT

#### Ureas

The adaptation of *Mtb* to adverse conditions such as hypoxia, acidic pH, and nutrient depletion entails a metabolic adjustment to slower or arrested replication and the inhibition of this adaptation is predicted to severely attenuate pathogenesis. The DosRST two-component regulatory system, consisting of two sensor histidine kinases, DosS and DosT, and the cognate response regulator DosR, regulates the adaptive response to hypoxia *in vitro* and *in vivo* with the associated metabolic downregulation resulting in a state of antibiotic tolerance. Inhibition of this process is predicted not only to affect persistence *in vivo* but also to increase vulnerability to drug regimens.

To identify inhibitors of the DosRST regulon, Zheng and co-workers executed a screen of a 540K library against *Mtb* where expression of a fluorescent protein under hypoxia was driven by a DosR-dependent promoter (Zheng et al., 2017). The selected hit, a diaryl urea derivative **64** (**Table 4**) (EC_50_ = 2.5 µM) was shown to directly bind to the heme of DosS and synergized with artemisinin which covalently modifies DosS. Mutations in DosS conferred compound resistant and transcriptional profiling revealed that **64** downregulated genes of the DosRST regulon.

To explore the SAR of urea **64**, commercially available and synthetic urea derivatives were used to understand the structural features of the series. Modification of A with different substituents on the phenyl ring or replacement with heteroaryl groups, was tolerated. In contrast, modifications to the central urea (B) by methylation, conformational restriction with another ring, or replacement with methylene reduced *Mtb* potency. The isoxazole (C) was critical for function since it could not be replaced with other aryls. Overall, **65** (**Table 4**) was the best analog with whole-cell DosRST inhibition (EC_50_ = 0.54 µM). Active analogs including **65** showed good kinetic solubility and favorable mouse microsomal stability, but PK studies and *in vivo* efficacy testing in a relevant animal are needed to validate the target.

## Conclusions

Drug development for TB remains a challenging enterprise but the entry of several molecules into clinical development highlighted here show that current efforts are yielding promising results after decades of lack of progress. Some common important lessons emerged during this review of the past ten years of effort: (1) pairing structure-based with whole cell read-outs was found many times to yield important discrepancies, often perhaps attributed to metabolism of certain scaffolds by *Mtb* cells, (2) cell-based activity is often increased by increasing lipophilicity within a scaffold to the detriment of the physicochemical properties and often resulting in stalling of hit series, (3) the main reason for failure of a series to progress was lack of efficacy in murine models of disease that often poorly resemble human disease, a serious risk to ever developing novel agents that might have game-changing properties in human use (Dartois and Barry, 2013). Finally, the progress highlighted here reiterates the fundamental importance of the screening conditions used and how they relate to human disease as we understand it, the uncertainties in the biology of TB disease seriously complicates medicinal chemistry efforts to develop novel treatments. The sobering reality is that progressing into human clinical trials is the only real validation of a mechanism of action that matters.

## Author Contributions

All authors contributed to background literature research and writing of the manuscript. All authors contributed to the article and approved the submitted version.

## Funding

This work was funded, in part, by the Intramural Research Program of the NIAID / NIH, and by the Bill and Melinda Gates Foundation through the TB Drug Accelerator program.

## Conflict of Interest

The authors declare that the research was conducted in the absence of any commercial or financial relationships that could be construed as a potential conflict of interest.
